# Conformational dynamics of androgen receptors bound to agonists and antagonists

**DOI:** 10.1038/s41598-021-94707-2

**Published:** 2021-08-05

**Authors:** Hyo Jin Gim, Jiyong Park, Michael E. Jung, K. N. Houk

**Affiliations:** Department of Chemistry and Biochemistry, University of California, Los Angeles, CA 90095-1569 USA

**Keywords:** Biophysics, Drug discovery, Chemistry

## Abstract

The androgen receptor (AR) is critical in the progression of prostate cancer (PCa). Small molecule antagonists that bind to the ligand binding domain (LBD) of the AR have been successful in treating PCa. However, the structural basis by which the AR antagonists manifest their therapeutic efficacy remains unclear, due to the lack of detailed structural information of the AR bound to the antagonists. We have performed accelerated molecular dynamics (aMD) simulations of LBDs bound to a set of ligands including a natural substrate (dihydrotestosterone), an agonist (RU59063) and three antagonists (bicalutamide, enzalutamide and apalutamide) as well as in the absence of ligand (*apo*). We show that the binding of AR antagonists at the substrate binding pocket alter the dynamic fluctuations of H12, thereby disrupting the structural integrity of the agonistic conformation of AR. Two antagonists, enzalutamide and apalutamide, induce considerable structural changes to the agonist conformation of LBD, when bound close to H12 of AR LBD. When the antagonists bind to the pocket with different orientations having close contact with H11, no significant conformational changes were observed, suggesting the AR remains in the functionally activated (agonistic) state. The simulations on a drug resistance mutant F876L bound to enzalutamide demonstrated that the mutation stabilizes the agonistic conformation of AR LBD, which compromises the efficacy of the antagonists. Principal component analysis (PCA) of the structural fluctuations shows that the binding of enzalutamide and apalutamide induce conformational fluctuations in the AR, which are markedly different from those caused by the agonist as well as another antagonist, bicalutamide. These fluctuations could only be observed with the use of aMD.

## Introduction

Androgen Receptor (AR) is a ligand-activated transcription factor that regulates the expression of many target genes that are crucial for male sexual differentiation and development. The AR plays a critical role in the development and proliferation of prostate cancer (PCa)^1,2^. In the early phase of PCa, androgen ablation therapy or chemotherapy with antiandrogens are established as the standard therapies. However, castration-resistant prostate cancer (CRPC) occurs after a few years of the standard treatment, which becomes more aggressive and ultimately lethal^3–5^. CRPC is still predominantly dependent upon the AR signaling pathway for its progression. The cellular mechanisms to resist the existing therapies include the AR gene amplification, the AR mutations, the androgen-independent AR activation and the appearance of AR splice variants^6^. The second generation AR antagonists, enzalutamide (brand name: Xtandi) and apalutamide (brand name: Erleada) are approved for clinical use in CRPC, which show much improved therapeutic efficacy over the first generation antagonists^4,7,8^.

The AR, a subfamily of nuclear receptors (NRs), consists of two well-defined structural domains, a DNA binding domain and a C-terminal ligand binding domain (LBD); there is also a highly variable N-terminal domain. The LBD has been a major interest in the drug discovery efforts, where most ligands and cofactors bind to^9^. Upon binding of a ligand to the ligand binding pocket in the LBD, NRs undergo conformational changes that are associated with the activation function-2 (AF-2). Biochemical evidence suggests that upon binding of an agonist such as testosterone, the repositioning of H12 and the formation of hydrophobic cleft follow, that promote the interaction of the AR with the LXXLL motif of co-activators^10,11^. On the contrary, the binding of antagonists abolishes the recruitment of co-activators by preventing H12 from folding over into the space that facilitates AR interactions with co-activators. The structural details of several NR antagonists bound to the LBD of other NR have been reported. An estrogen receptor alpha (ERα) antagonist, raloxifene, induces repositioning of H12 by its bulky side chain and disrupts the overall surface topography of AF-2^12^. Despite many attempts, the structural basis of the AR antagonism is not fully understood. There are over 90 x-ray crystal structures of the human AR LBD deposited in Protein Data Bank. However, all of the existing crystal structures represent functionally activated ARs either wild type bound to agonists or drug-resistant mutant ARs bound to antagonists (Table 1)^13^.

**Table 1:** X–ray crystal structures of AR LBD from Protein Data Bank and their 3D structural alignment complexed with various ligands.

PDB ID	AR type	Ligand	
1XQ3	Wild type	AR agonist (R1881)^14^	
1T63	Wild type	AR agonist (dihydrotestosterone; DHT)^15^	
2AX6	T877A mutant	AR antagonist (hydroxyflutamide)^16^	
2AXA	Wild type	AR agonist (S1)^16^	
1Z95	W741L mutant	AR antagonist (R-bicalutamide)^17^	
2OZ7	T877A mutant	AR antagonist (cyproterone acetate)^18^	
2PIP	Wild type	AR agonist (DTH)/BF3 inhibitor (K10)^19^	
3V49	Wild type	SARM (diarylhydantoins)^20^	
5JJM	Wild type	AR agonist (DHT)^21^	
5VO4	Wild type	SARM (pyrrole-2-carbonitrile derivatives)^22^	

The key mutant residues (W741, T877 and F876) near the binding site for the ligands are represented as colored in blue. Image was created by using Visual Molecular Dynamics (VMD) v1.9.3^52^.

Computational studies have elucidated the molecular basis of AR antagonism. Bisson et al*.* proposed that the sulfonyl group of R-bicalutamide pushes W741 from its activating position, thereby achieving the observed antagonism. They also showed that the decreased activity of hydroxyflutamide (HFT) on wild type AR is caused by the weaker interaction between HFT and Met895 compared to T877A mutant AR^23^. Zhou and co-workers employed replica exchange molecular dynamics (REMD) simulations to study the structural changes of the WT and the T877A mutant ARs bound to HFT. Conformations of the H12 sampled from the REMD simulations were shown to have a correlation with the agonistic/antagonistic activities of the ligand^24^. By performing MD simulations and quantum mechanics (QM) calculations, Osguthorpe and Hagler demonstrated that bicalutamide can access an additional binding pocket (B-site) of the AR LBD, which results in the instability of H12^25^. Based on 10 ns MD simulations, Balbas et al*.* showed F876L mutation in AR allows enzalutamide to act as an agonist^26^. Xu et al*.* analyzed the structural stability of AR upon binding of dihydrotestosterone (DHT) and co-activator (SRC), based on 20 ns MD simulations^27^. Liu et al*.* demonstrated that the ligand binding affects the AR–coactivator interactions as well as the allosteric regulation pathway from ligands to co-activator, based on MD simulations and free energy calculations^28^. By using homology modeling, Wang et al*.* constructed a putative antagonistic structure of the AR and proposed the role of H12 in the molecular function of the AR^29^. Duan et al*.* carried out microsecond unbiased MD simulations as well as bias-exchange metadynamics MD simulations to quantify the conformational characteristics of antagonist bound AR LBD^30^. Recently, Hu et al. summarized the recent advances in the discovery of AR antagonists using computer-aided drug design approaches^31^. The computational analyses comprehensively support the mechanistic hypothesis that the AR antagonists cause conformational changes to the AR LBD, which can be correlated with the antagonism of the AR. However, to our best knowledge, the influences of the second-generation antagonists such as enzalutamide and apalutamide to the conformational dynamics of the AR LBDs have not been elucidated in detail. Moreover, a comprehensive comparison of the conformational changes in AR LBDs bound to the agonists, the first- and the second-generation antagonists has not been carried out. In addition, a structure activity-relationship that is readily applicable to the discovery of novel AR antagonists is in dire need for the rapid screening of next-generation AR antagonists.

Herein, we examined the detailed structural changes of the AR LBDs caused by the binding of antagonists by means of computational modeling and molecular dynamics (MD) simulations of the various ligands bound to the AR LBD. We have studied conformational dynamics on the LBDs of the wild type and F876L mutant AR in the presence of the first and the second-generation antagonists, the agonists, and in the absence of ligands (*apo*) using accelerated MD simulations. We found that the binding of agonists stabilizes the AR LBDs by retaining the agonistic conformation, that are in agreement with the previous experimental and computational results. The simulations revealed that the wild type AR bound to antagonist showed distinctive conformational dynamics depending on the initial pose and the nature of the antagonists. We explored two distinct binding poses of the N-methylbenzamide moiety of enzalutamide in the AR. The results show that the structural changes of α-helix 12 (H12) only occurs when the N-methylbenzamide of enzalutamide binds proximal to the H12, but not when it directs toward α-helix 11 (H11). We also found that the F876L mutation of the AR stabilizes the agonistic conformation, regardless of the conformation of enzalutamide in the binding pocket. Binding of bicalutamide to the wild type AR destabilizes the H1–H3 loop significantly, unlike the case of enzalutamide. The comparison of two distinct conformational changes induced by bicalutamide and enzalutamide suggested two different mode of antagonistic actions, in spite of their structural similarities. The finding suggested a new mechanistic scenario for AR antagonism caused by the second-generation antagonists, where a large conformational change in H12 directly hampers the binding of the cofactors to AR LBDs. Finally, we performed principal component analysis (PCA) on the conformational dynamics of the AR in the presence of agonists, antagonists, and the absence of ligand. The analysis suggested that one can correlate the efficacy of each ligand with the conformational fluctuation of LBD sampled from aMD simulations.

## Results and discussion

### Structures of AR ligands in the ligand binding domain of AR

**Figure 1:**
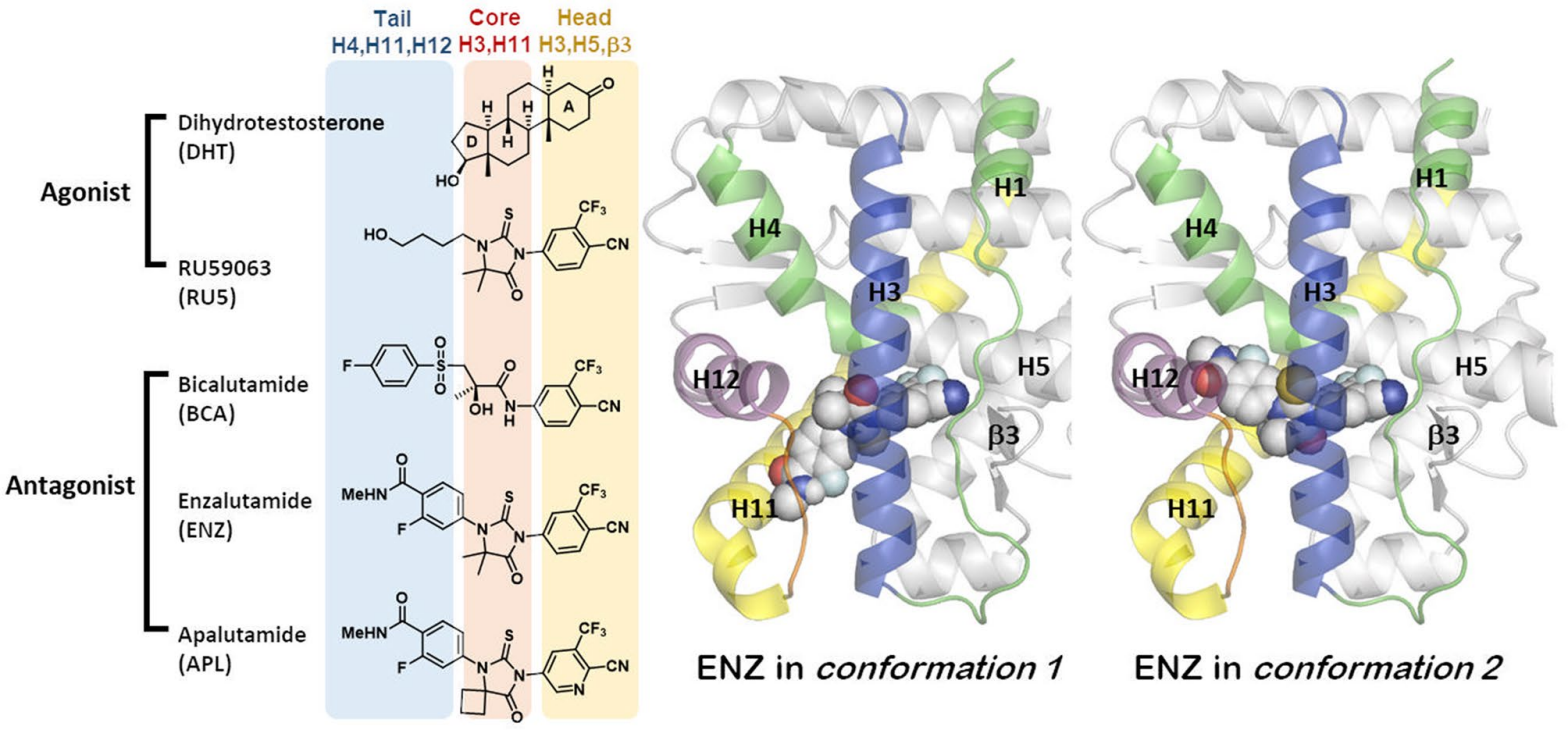
Representative AR agonists and antagonists. Two proposed binding conformations for enzalutamide in AR LBD. Images were created by using Chemdraw and VMD v1.9.3^52^.

Figure 1 shows the chemical structures of the five AR ligands studied here. Each represents a specific class of ligands: dihydrotestosterone (DHT) is a natural agonist and RU59063 (RU5) is an agonist and the lead compound we used for the development of enzalutamide and apalutamide. R-Bicalutamide (BCA), enzalutamide (ENZ) and apalutamide (APL) are AR antagonists, approved for therapeutic application by the FDA. APL has a similar chemical structure to ENZ, but shows a greater efficacy and a higher therapeutic index with lower steady-state plasma levels than ENZ^32^. The chemical structures of the ligands can be divided into three structural units that determine the binding positions. The C3 ketone on the A-ring of DHT is essential for the AR binding by interacting with the key residues on H3, H5 and β3 of the AR LBD. A number of non-steroidal AR agonists or antagonists replace the A-ring of DHT with substituted aromatic rings like a 3-trifluoromethyl-4-cyanophenyl (Fig. 1) or a 3-chloro-4-cyanophenyl group. The central parts of the structures occupy the ligand binding pocket covered with H3, H4 and H11 and control the shape of the ligand. The left-most segment plays a key role to produce agonist or antagonist properties by stabilizing or disrupting H12 (vide infra).

We initially carried out 250 ns conventional MD simulations with the bound AR ligands. The backbone root-mean-square deviation (RMSD) variation of the AR bound to ENZ was small (< 2 Å), and there were no notable changes from the starting agonistic conformation (Figure S1). As such, we applied accelerated MD simulations to observe large-scale conformational dynamics of the LBDs in apo and bound to the ligands of interest^33^. Accelerated MD (aMD) is a method to greatly enhance conformational sampling of biomolecules. Using aMD can facilitate conformational transitions of proteins over 500-fold than the conventional MD simulations. The aMD method has been successfully applied to study conformational dynamics of proteins and protein–ligand complexes^34–36^. We monitored the conformational changes of the AR LBD, particularly the pockets near H11 and H12 as well as the cofactor binding region near the ligand binding pocket during aMD simulations. To analyze further the structural fluctuations of the AR LBD simulated, principal component analyses (PCA) were employed to examine the relationships of conformations observed when agonists and antagonists bind to the AR^37,38^.

Table 2 summarizes the systems which have been simulated with aMD. Two PDB structures (2AXA and 2PIP) were chosen as initial structures for the simulations. Both of the PDB structures represent agonistic conformations of the wild type (WT) AR, which are co-crystallized with a synthetic agonist S1^16^ and DHT^19^. The ligands were removed for the simulations of *apo* AR. We conducted constant temperature MD simulations of 250 ns duration and then commenced aMD simulations for 250 ns, starting from the last structure of the cMD simulations (see SI for details).

**Table 2:** Model systems simulated with aMD in this study.

PDBs:2AXA, 2PIP	Wild type AR	Mutant AR
*apo*	No ligand	No ligand
Agonist	DHT	–
	RU59063^a^	–
Antagonist	ENZ^a^	(W876L) ENZ^a^
	APL^a^	–
	BCA^a^	(W741L) BCA

^a^The simulations were performed with the ligands in both *conformation 1* and *2.*

### Accelerated MD simulations of AR in apo and AR bound to agonists

**Figure 2:**
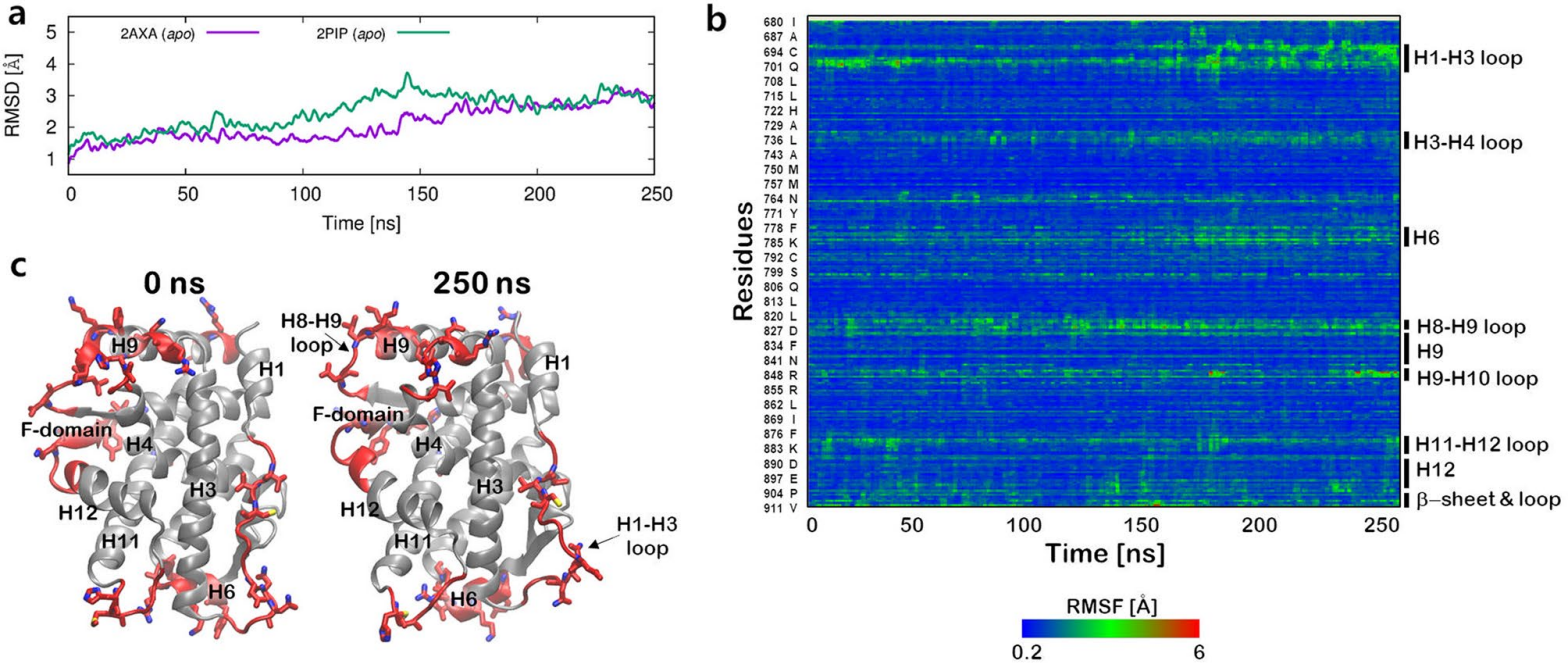
Conformational changes of *apo* AR during 250 ns aMD simulations. (**a**) The backbone RMSD. (**b**) RMSF analysis. (**c**) The comparison of the initial X-ray crystal structure of the AR (PDB ID: 2AXA) after removal of the ligand (S1) and the resulting structure after 250 ns aMD simulation. The regions showing RMSF > 2.5 Å are colored in red. Images were created by using VMD v1.9.3, plugin^52^ and Gnuplot v5.2^57^.

Figure 2 summarizes the aMD simulations of AR in *apo*. Figure 2a shows the backbone RMSDs of the simulated *apo* AR LBDs from the initial X-ray crystal structures. For both of the aMD trajectories initiated from the two PDB structures, the backbone RMSDs gradually increase to 3.0 Å after 250 ns. In the absence of ligands, both PDB structures undergo structural changes from the initial agonist-bound conformation. To examine residue specific atomistic fluctuations, root mean square fluctuations (RMSF) were calculated after superimposing all the trajectories to the initial conformation (Fig. 2b). We observed significant RMSF changes in a loop region H1–H3, H8–H9 and H9–H10. In particular, a series of movement of several amino acids (P682 to D695, colored in red in Fig. 2c) on H1–H3 loop was observed. Although the functional role of the loop is not well understood in the AR, the H1–H3 loop in glucocorticoid receptor (GR) serves as the interaction for a heat-shock protein (Hsp90) and the co-chaperone complexes and influences the hormone binding affinity^39,40^. Due to the conformational similarity of LBD to the other steroid receptors, we speculate that this loop in the AR may also affect interactions with the chaperon machineries.

**Figure 3:**
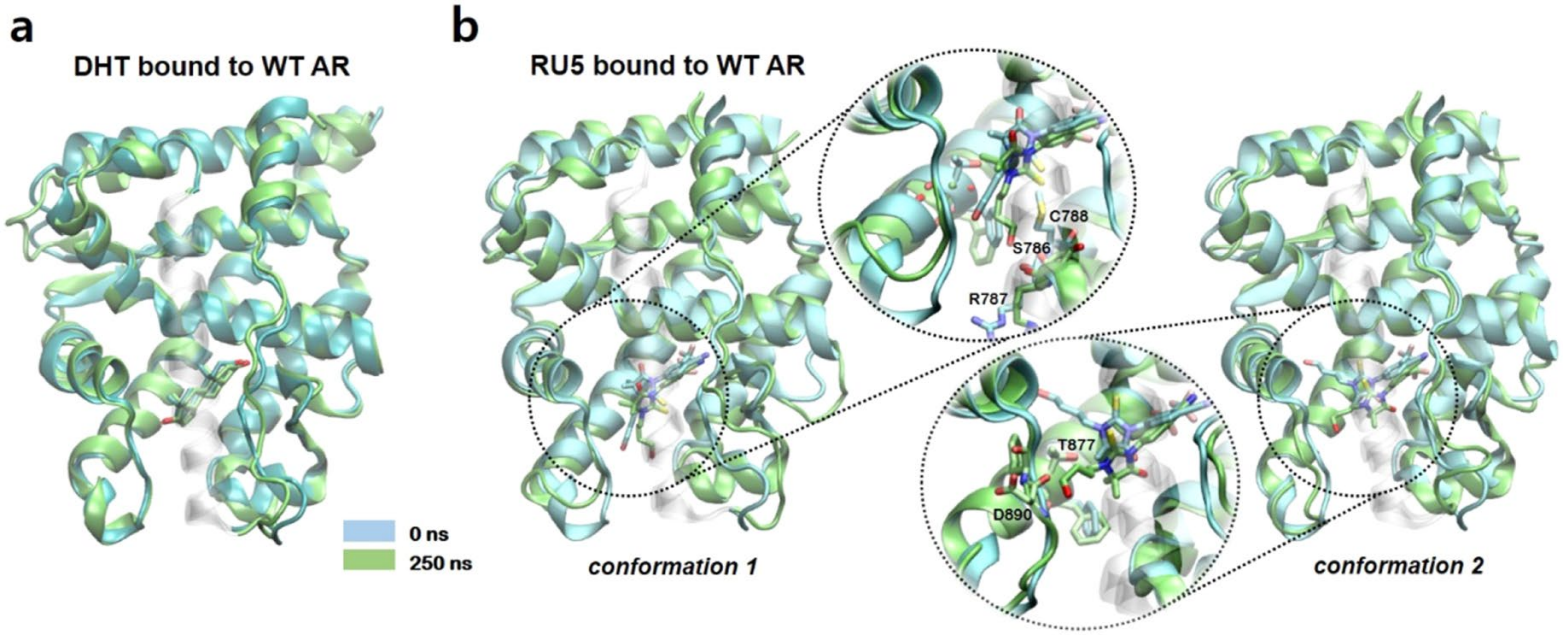
The wild type ARs bound to the agonists. (**a**) The snapshots of the initial structure (PDB ID: 2PIP) of the wild type ARs bound to DHT and the resulting structure after 250 ns aMD simulations. Initial and 250 ns simulated structures are colored in cyan and lime, respectively. (**b**) The wild type ARs bound to RU5s. Initial structures and 250 ns aMD simulated structures are colored in cyan and tan, respectively. Images were created by using VMD v1.9.3^52^.

The binding of a natural substrate (DHT) and a synthetic agonist (RU5) maintained the agonistic conformation after 250 ns of aMD simulations (Figs. 3 and S2). Upon binding to DHT, the RMSD changes in the AR did not exceed 3.0 Å from the initial conformations that represent agonistic conformation of the AR (Figs. 3a and S2B). The binding of RU5 retained the structural integrity of the AR LBD (Figs. 3b and S2C). Of note, two possible orientations of thiohydantoin group of RU5 were considered (*conformation 1* and *2* in Fig. 3b). However, the RMSD from the initial agonistic conformation did not exceed 2.5 Å through most of the simulation period. These results demonstrate that the binding of agonists stabilize the AR LBD by holding the structural components that surround the ligand binding pocket, when compared with the structural changes that were observed from the AR in *apo.* In the following, we demonstrate that the AR LBD becomes unstable upon binding to the antagonists of AR.

### Binding of ENZ displaces H12 of the AR

**Figure 4:**
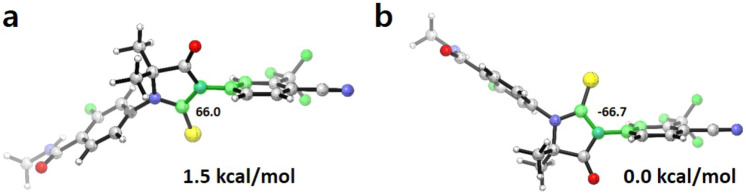
Two lowest energy conformers of ENZ. (**a**) Conformer with the second lowest in energy (*conformation 1*) and (**b**) the lowest in energy conformation (*conformation 2*). The dihedral angles of the four highlighted atoms (C-C-N-C) are shown. Images were created by using CYLView v.1.0.561^56^.

**Figure 5:**
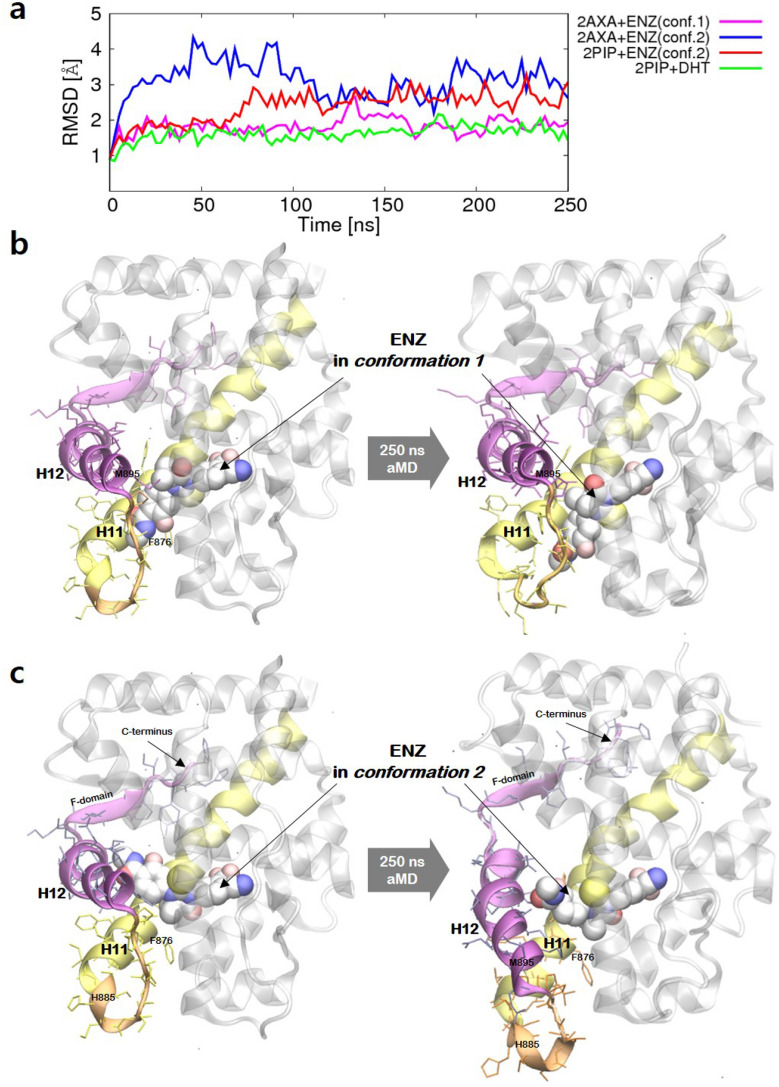
Conformational changes of the wild type AR bound to ENZ during 250 ns aMD simulations. (**a**) The backbone RMSDs of AR-ENZ complexes relative to the initial agonist conformation. The visualizations of initial and 250 ns simulated structures of the wild type AR bound to ENZ in (**b**) *conformation 1* and (**c**) *conformation 2*. Images were created by using Gnuplot v5.2^57^ and VMD v1.9.3^52^.

We examined plausible binding modes of ENZ in the ligand binding pocket of AR, as high resolution structural information of the AR LBD bound to ENZ is not available. In the beginning, we explored viable conformers of ENZ by means of quantum mechanical calculations. As the head group (benzonitrile) and the core group (thiohydantoin) can rotate across the C–N bond connecting the two functional moieties, multiple energetic minimums were expected. Our quantum chemical calculations demonstrated that the two preferred conformers differ in Gibbs free energy by 1.5 kcal/mol (Fig. 4), suggesting that both of the conformers are accessible in vivo. The two conformers are termed as *conformation 1* and *conformation 2*, respectively. Starting from the two conformers, we explored the possible conformations of ENZ in the binding pocket of AR LBD based on the molecular docking simulations and the top scored binding pose of ENZ was selected as a starting structure for MD simulations. For *conformation 1*, the N-methylbenzamide tail stretches toward H11 (Fig. 5b). For *conformation 2*, the tail of ENZ is placed in the space between H4 and H12 (Fig. 5c). The pose of the tail group in *conformation 2* resembles the conformation of the tail group of an agonist (S1) that was co-crystallized with the AR LBD^16^ and that of BCA proposed by Osguthorpe and Hagler^25^. In the following, we demonstrate that the binding of ENZ in *conformation 2* induces conformational changes in the surrounding regions of the binding pocket of AR LBD.

Figure 5 summarizes aMD simulations of the wild type AR LBDs bound to the two distinct conformers of ENZ. When ENZ is bound in *conformation 2* large conformational changes of the AR occur as shown in Fig. 5a,c. The backbone RMSDs from an initial structure (PDB ID: 2AXA) with ENZ in *conformation 2* increased to > 3 Å after 10 ns and reached to 4.5 Å in 50 ns. From aMD simulations using another crystal structure of AR LBD (PDB code: 2PIP), we also found the backbone RMSD increased to 2.5 Å (Fig. 5a), when ENZ is bound in *conformation 2*. The RMSD changes of *conformation 1* compared to the initial structure were not as dramatic as, the value remained < 2.5 Å throughout the simulations, except for 130 ns to 140 ns (Fig. 5a). The magnitude of RMSD change was comparable to those of DHT or RU5 bound to AR. The results suggested that *conformation 2*, where the tail group of ENZ orients toward H12, can disturb the structural stability of the agonistic conformation.

Ensemble averaging of the MD and the aMD simulations confirmed the statistical significance of the increased backbone RMSD in the AR LBD bound to ENZ in *conformation 2*. Figure S11 summarizes the distributions of backbone RMSDs, measured from the single 250 ns MD and aMD trajectories and the ensembles of 5-independent 250 ns MD and aMD trajectories. For the conventional MD simulations, the distributions were centered at 1–1.5 Å, suggesting minor conformational changes in the agonistic conformation, regardless of the number of independent MD trajectories considered. For the aMD simulations of AR LBDs bound to ENZ in *conformation 2*, we observed right-shift in the distribution maximum relative to that of ENZ in *conformation 1*. Of note, the right-shifts in the maximum observed from the ensemble-averaged distributions of ENZ in *conformation 2* were less pronounced than those computed from single 250 ns aMD trajectories. The observation suggested that ensemble averaging of conformational changes based on multiple independent aMD simulations is necessary for prognostic aMD simulations, where one aims at predicting the efficacy of ligand prior to experimental tests.

Figure 5b,c compare the initial and the final structures of the wild type AR bound to ENZ, sampled from 250 ns aMD simulations (PDB code: 2AXA). From the aMD simulations with *conformation 2*, we observed significant structural differences arose from the repositioning of H12 and connecting loop between H11 and H12. The Cα RMSF over the time indicated that a number of residues, including H885 to M895 on H11–H12, loop over H12 and the residues (K912 to H917) in the end of C-terminus, showed significant fluctuations in the course of aMD simulations (Figures S3 and S4). The increment in RMSD and RMSF correlated with the tilt in H12 relative to H11. We measured the relative angle between H11 and H12 from the MD and the aMD trajectories of AR bound to ENZ (Figure S12). From the aMD simulations using 2AXA bound to ENZ in *conformation 2*, the angle decreased from 70° to 51° in 20 ns, that complies with the increment of backbone RMSD to > 3.0 Å in 10 ns. From the aMD simulations of ENZ in *conformation 2* bound to another agonistic conformation of AR (PDB code: 2PIP), the angle began to decrease to 50° after 70 ns from the initiation of simulations, that coincided with the onset of increment in RMSD to > 3.0 Å.

**Figure 6:**
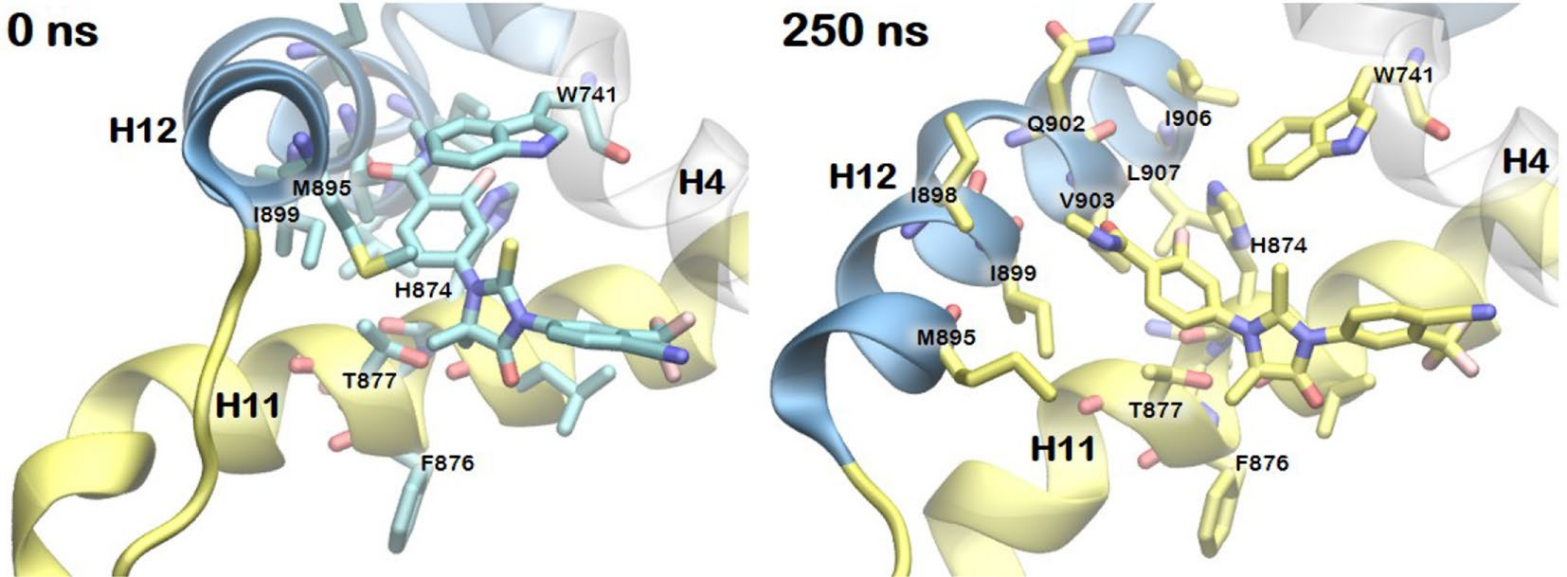
Representative snapshots for the detailed binding mode of ENZ in *conformation 2* in the AR LBD. The initial structures of ENZ and key residue are colored in cyan and the simulated structures are colored in yellow. Images were created by using VMD v1.9.3^52^.

Figure 6 highlights the regions in the AR LBD that show significant conformational changes upon binding of ENZ in *conformation 2*. In the initial agonistic structure, H12 is located in the close vicinity of the ligand binding pocket surrounding H4 and H11 (Cα distances of 7–11 Å from H4). During the aMD simulation, the N-methylbenzamide moiety of ENZ extends toward H12 that results in a large displacement of the helix from the initial position that covers the ligand binding pocket (Cα distances of 9–15 Å from H4). The bulky and hydrophobic side chains of H12 (M895, L898, V903, I906 and L907) facing the binding pocket were influenced by ENZ. Repositioning of H12 causes a dynamic movement of the H11–H12 loop and displacement of F-domain composed of β-sheet as well as the flexible loop. In short, the binding of ENZ in *conformation 2* repositions H12 by repulsive interactions with the bulky and hydrophobic side chains on H12.

### F876L stabilizes AR LBD bound to ENZ

**Figure 7:**
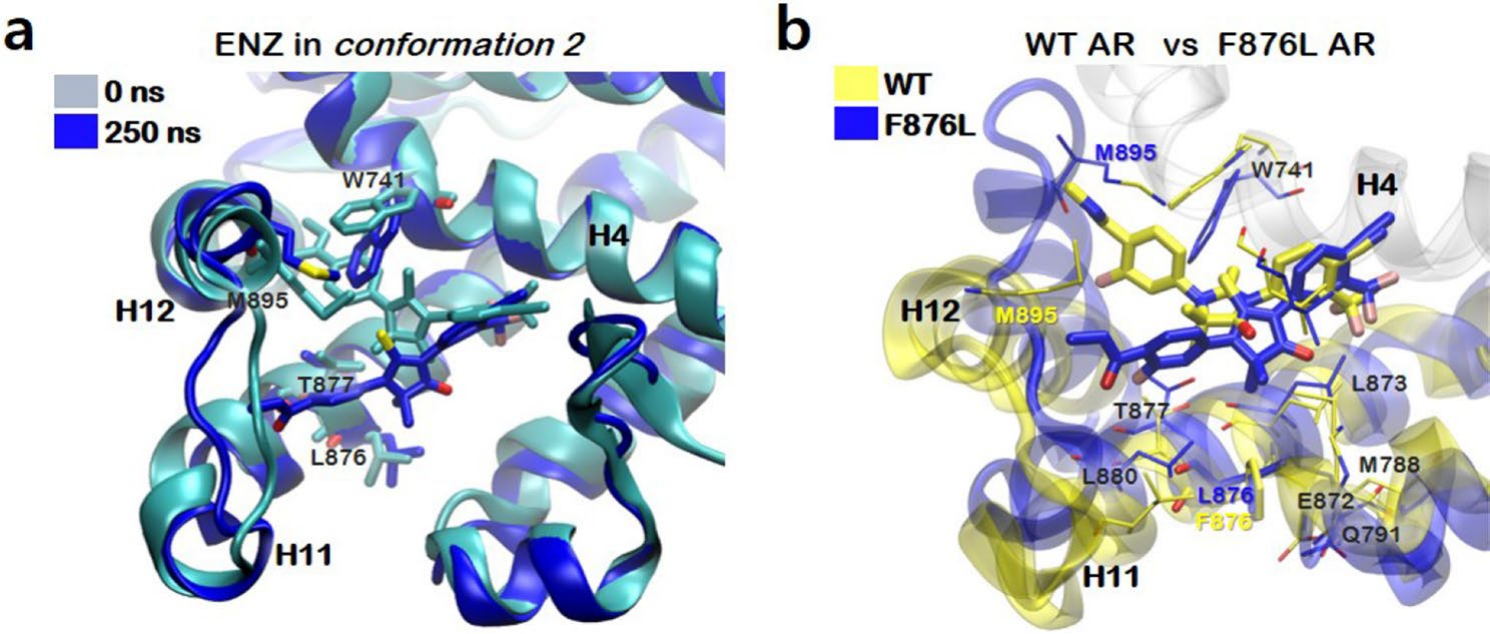
Conformational dynamics of the F876L mutant AR bound to ENZ in *conformation 2*. (**a**) Comparison of the ligand binding pocket at 0 and 250 ns. Initial structures are colored in cyan and simulated structures with ENZ in *conformation 2* are colored in blue. (**b**) Comparison of the ligand binding pocket of the wild type (yellow) and F876L mutant ARs (blue) bound to ENZ in *conformation 2* after 250 ns aMD simulation. Images were created by using VMD v1.9.3^52^.

Point mutations in the ligand binding pocket of AR can lead to the drug resistance by converting antagonists into agonists. T877A, W741L, F876L are the most prevalent drug resistant mutations for HFT (the active form of flutamide), BCA and ENZ, respectively^41–43^. We performed aMD simulations of the AR F876L mutant in the presence of ENZ in *conformation 1* and *2* and in the absence of the ligands, as to understand the origin of drug resistance caused by the mutation. For all simulations, we observed RMSDs < 2.5 Å from the initial agonistic structure (Figure S5A). Unlike the wild type *apo* AR, F876L mutant in *apo* state was stable during 250 ns simulations without any significant conformational changes (Figure S5B). The binding of ENZ in *conformation 1* to the mutant AR stabilizes the agonistic conformation, similarly to ENZ in *conformation 1* (Figure S5C). Interestingly, the N-methylbenzamide tail of ENZ in *conformation 2* moves into the space along H11 without disturbing H12 after 200 ns (Fig. 7a).

An analysis of the ligand binding pocket of AR F876L suggested that the mutation reduces steric demand between H11, H12, and ligand. First we observed that, when averaged over 250 ns of constant temperature MD simulations, the volume of the ligand binding pocket of the F876L mutant was 101 ± 30 Å^3^, which is 36% larger than that of the wild type AR (74 ± 26 Å^3^), which were measured by POcket Volume MEasurer (POVME) program^44^. The increased volume may reduce the steric demand to accommodate the tail group of ENZ in *conformation 2*. In the F876L mutant, the gem-dimethyl of ENZ in *conformation 2* is placed toward H3, H11 and the end of H6, resulting in a repositioning of benzamide tail of ENZ into H11 (Fig. 7b). By contrast, in the wild type AR the F876 residue precludes the gem-dimethyl of ENZ from approaching the space crowded with bulky side chains of M788, F705 and L709. These results demonstrate that the F876L mutant AR bound to ENZ is close to the agonistic conformation of the AR regardless of the binding of ENZ.

### Binding of APL repositions H6 and H11 as well as H12

**Figure 8:**
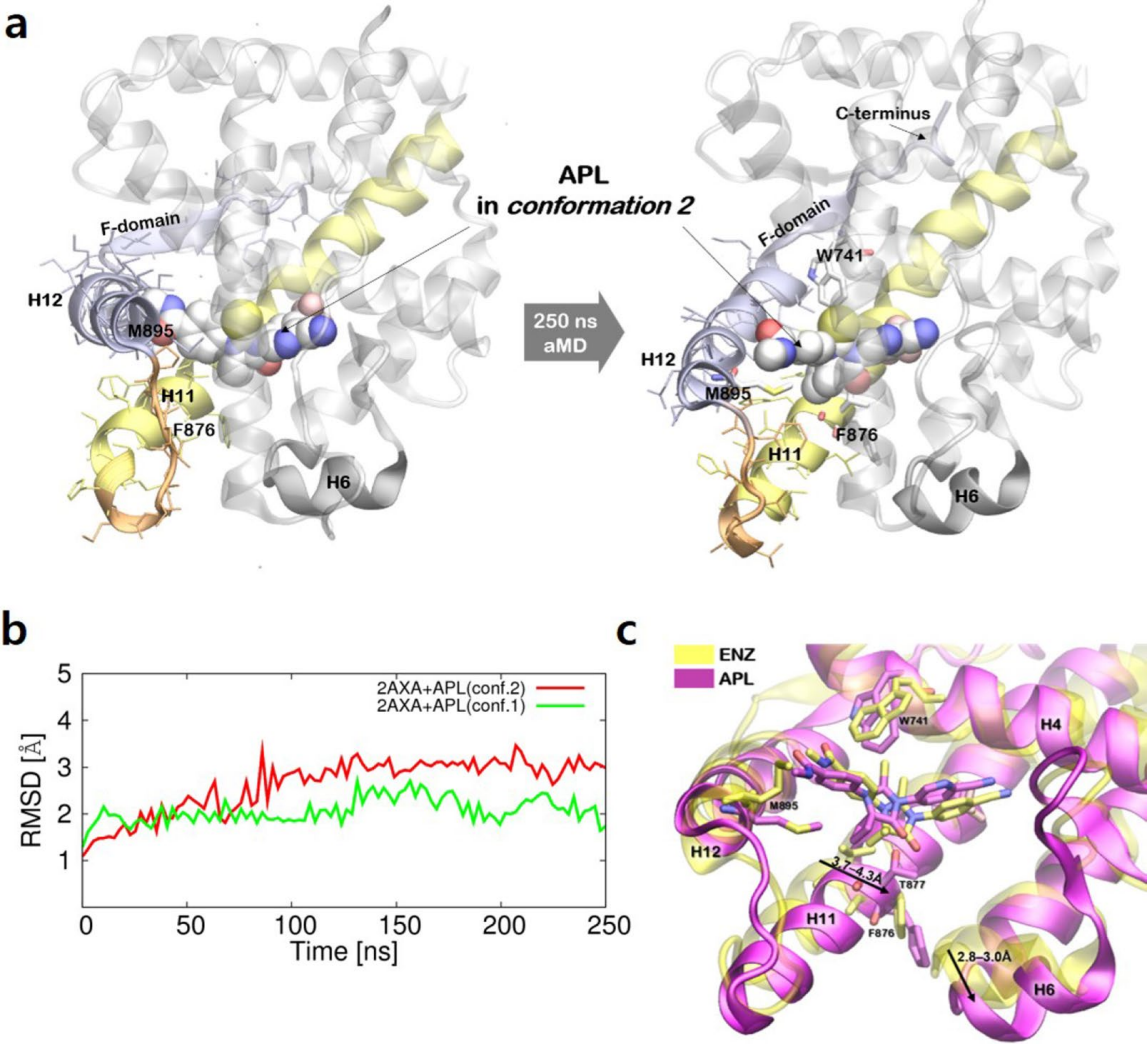
Conformational changes of the wild type AR bound to APL in *conformation 2*. (**a**) Visualizations of 0 and 250 ns aMD simulations. (**b**) The backbone RMSDs. (**c**) Comparison of the ligand binding pocket of the simulated AR bound to ENZ (yellow) and APL (magenta) in *conformation 2* after 250 ns. Images were created by using VMD v1.9.3^52^ and Gnuplot v5.2^57^.

Figure 8 represents conformational changes of the wild type AR bound to APL in *conformation 2* during 250 ns aMD simulation. The structural differences between APL and ENZ are only the pyridine ring in the head and cyclobutyl moiety instead of dimethyl group on the thiohydantoin core. We wondered how this subtle structural difference affects the conformation of the AR LBD. We initially explored the conformers of APL by carrying out QM calculation. Two conformers having distinct orientation between the pyridyl head group and the thiohydantoin group differ in Gibbs free energy by 1.9 kcal/mol (Figure S6), suggesting that the two conformers are viable in solution. Therefore, we considered the two conformations as the starting point at the simulations, as in the case of ENZ. Our aMD simulations showed that only APL in *conformation 2* perturbs initial agonistic conformation by repositioning H12 and H11–H12 connecting loop in much the same way as ENZ (Figs. 8a,b and S7). H12 began to move away from the ligand binding pocket after 100 ns, resulting in RMSD from the initial conformation greater than 3 Å. More importantly, in addition to the repositioning of H12, we observed additional movement of H11 and H6 the wild type AR bound to APL (Fig. 8c). We correlated these structural changes to the presence of cyclobutyl moiety in the core group of APL. The Cα RMSF over the time indicated that the residues V692-C694 and N699-Q701 on H1–H3 loop, Y781-R787 on H6 and H885-M895 on H11–H12 loop and H12 were significantly fluctuated during aMD simulations (Figure S7). We note that no hydrogen bonding interactions between the nitrogen on pyridine and amino acids of the ligand binding pocket were observed during simulations, suggesting that replacement of phenyl ring of ENZ with pyridine does not significantly affect the structural changes of AR caused by APL. In short, we demonstrated that the tail group shared by ENZ and APL induces structural fluctuations in H12 and the introduction of cyclobutyl moiety at the thiohydantoin core can induce additional structural changes in H11, which may explain increased therapeutic efficacy of APL over ENZ.

### BCA induces conformational changes in the H1–H3 loop and the H3 of AR

Bicalutamide (BCA) is a nonsteroidal AR antagonist known as the predecessor of ENZ. BCA has 5–8 times lower affinity to the AR than ENZ despite the structural similarity of the head group. The AR inhibitory mechanisms of BCA is primarily the blocking the binding of androgens to AR^45^. Of note, ENZ impedes the nuclear translocation of AR, the binding of AR to DNA and the interactions of AR with the coactivators. Moreover, ENZ does not show agonistic effect on the wild type AR^7^.

**Figure 9:**
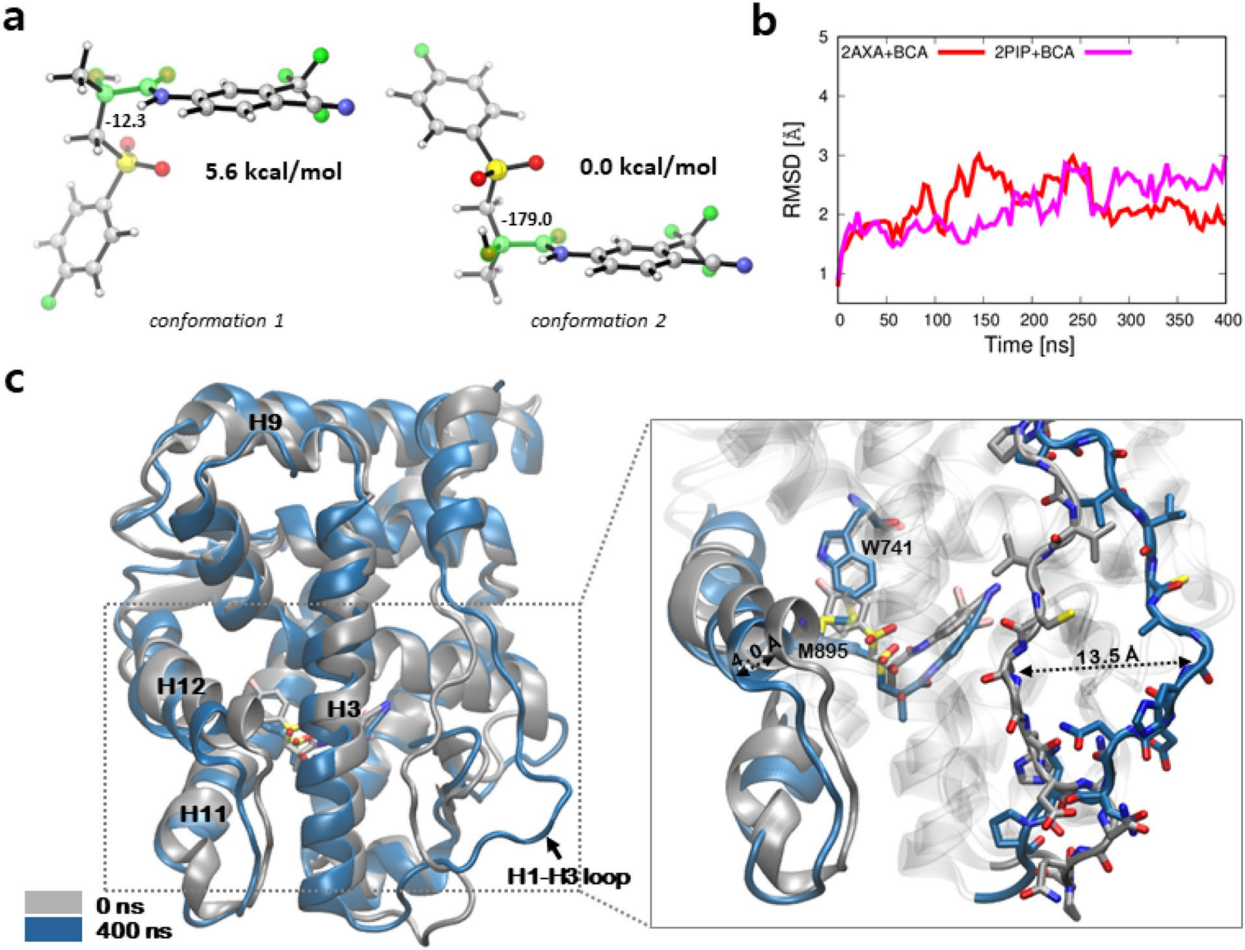
Comparison of conformational changes of the wild type AR bound to BCA. (**a**) Two conformers of BCA with the lowest energy; Left: *conformation 1* with the second lowest energy; Right: *conformation 2* with the lowest in energy conformation. The dihedral angles of the four highlighted atoms (O-C-C-O) are shown. Images were created by using CYLView v.1.0.561^56^. (**b**) The backbone RMSDs of aMD simulations relative to the initial conformation. Gnuplot v5.2^57^. (**c**) Comparison of the wild type ARs bound to BCA in *conformation 2* at the initial (silver) and simulated structure after 400 ns (blue).Images were created by using VMD v1.9.3^52^.

Figure 9a shows two of the viable conformations of BCA. We explored the structures and energies of conformations with quantum mechanical calculations and confirmed that BCA *conformation 2* is 5.6 kcal/mol lower in Gibbs free energy than *conformation 1*. Of note, S1, the parent compound of BCA, was co-crystallized with the AR with the 4-fluorobenzenesulfonyl group positioned toward H12^16^. These findings strongly suggested that BCA binds to the AR in a similar orientation to that of S1. As such, in the following MD simulations, we considered the conformation BCA in the binding pocket of AR, which resembles that of S1 (Figure S8A).

We compared the conformational dynamics of AR bound to BCA using a series of aMD simulations. We constructed models of AR bound to BCA using two of available x-ray structure of AR (PDB ID: 2AXA and 2PIP). Figure 9b shows the plot of backbone RMSDs of the aMD simulations. The RMSD fluctuations were less than 3 Å relative to the initial conformation even after 400 ns of aMD simulations, which was less pronounced than that of the AR bound to ENZ. Since the structural difference between S1 and BCA is minor (Fig. S8A), we thought that BCA may not induce large conformational changes relative to ENZ. An investigation on the sampled conformations revealed that there are small but notable conformational fluctuations in several loop regions in the AR bound to BCA (Fig. 9c). The comparison of the simulated conformation after 400 ns with the initial conformation, we realized that the loop connecting H1 and H3 (H1–H3 loop) moved outward and the part of H3 tilted toward the H11–H12, when compared to the AR bound to DHT or ENZ (Figure S8B). On the contrary, aMD simulations of a drug resistant mutant (W741L) bound to BCA showed that the H1–H3 and the H11–H12 loops are stable after 250 ns (Figure S9), suggesting the observed conformational changes in the WT AR bound BCA can be correlated with the action of the antagonist. The plot of RMSF indicates the conformational fluctuation of the residues on this region was significantly increased as well (Figure S8C). We found that H12 and the connecting loop also fluctuate in this system, but not as much as that of the AR bound to ENZ.

Based on our computational results, we concluded that the binding of BCA to the AR LBD inhibits the binding of androgens by inducing destabilization of H1–H3 loop and a tilt of H3 as well as nudging H12 outward, whereas ENZ and APL antagonize the AR by significantly displacing H12. The conformation of BCA is in a bent shape formed by chiral center bearing a methyl and a hydroxyl group, and a benzene sulfonyl moiety. By contrast, ENZ and APL have a thiohydantoin ring at the core that connects the benzyl (pyridyl) head and the benzamide tail group via two nitrogen atoms on the ring. The rigid core and extended tail of ENZ and APL enable the conformations of the ligands to maintain the linearity during the aMD simulations, which result in the repositioning of H12. From these observations, we speculate that the core of the two antagonists (ENZ and APL) can modulate conformational dynamics of the AR LBD, which can explain the improved efficacies of ENZ and APL to the wild type AR than BCA.

### PCA reveals distinct conformational fluctuation of the AR by the binding of ligands

**Figure 10:**
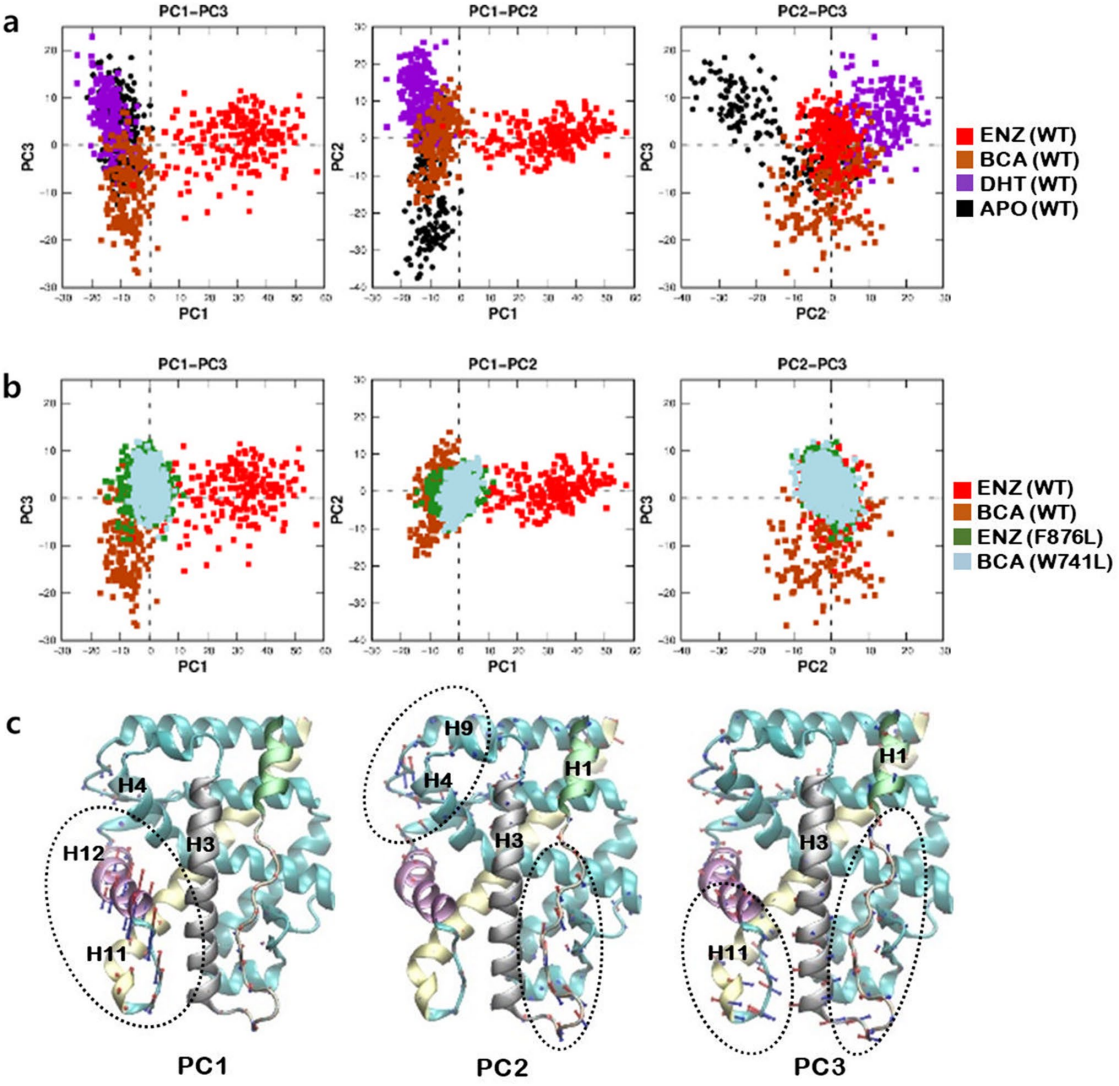
PCA of trajectories obtained from aMD simulations of the wild type ARs bound to the ligands. (**a**) Projections of sampled coordinates onto PC axes for the first three PCs. Each color indicates aMD trajectories of AR bound with ENZ in *conformation 2* (red), BCA (brown), DHT (purple) and no ligand (*apo*, black). (**b**) Projections of aMD trajectories of the wild type ARs bound to ENZ in *conformation 2* (red), BCA (brown), F876L mutant AR bound to ENZ in *conformation 2* (green) and W741L mutant AR bound to BCA (cyan) (**c**) Visualizations of the three PCs. Arrows show the direction and relative magnitude of Cα motions along the corresponding PC. Colors of arrows represent the positive (blue) and the negative (red) directions along each PC eigenvector. Images were created by using Gnuplot v5.2^57^ and VMD v1.9.3^52^.

Figure 10 summarizes the principal component analysis of the aMD trajectories of AR LBDs, which was performed to quantify the observed conformational fluctuations. We computed the principal components using the agglomerated aMD trajectories of the four aMD trajectories; AR LBDs bound to DHT, ENZ, BCA, and in *apo*. Prior to the computations of principal components, the backbone trajectories were aligned to an X-ray structure of the AR (PDB code: 2AXA), so as to remove the translation-rotational motions of the protein. We then calculated the projection of each aMD trajectory to the computed PCs, as shown in Fig. 10a. WT AR bound to ENZ showed a notable fluctuation along the first principal component (PC1); the conformational fluctuations of AR in *apo* were captured by the second principal component (PC2); the fluctuations of AR bound to BCA were characterized by the third principal component (PC3).

The projection of aMD trajectories of drug resistant mutants bound to antagonists confirmed that the mutations minimizes the conformational changes in the agonistic conformation of AR. Using the computed PCs based on the aMD simulations of WT AR, we projected the backbone trajectories of W741L bound to BCA and F876L bound to ENZ (Fig. 10b). The projection demonstrated that the drug resistant mutations reduce the characteristic structural fluctuations found from the WT AR bound to the antagonist.

The direction of the backbone dynamics in each PC is also informative. Figure 10c represents the direction of those fluctuations on the AR structures. In PC1, the positive motion (colored in blue) moves H12 and the connecting loop away from the ligand binding pocket formed by H3, H4, and H11, which strongly supports the proposition that the repositioning of H12 is one of the key antagonistic mechanisms. In PC2, the dominant fluctuations occur at the flexible H1–H3 and H8–H9 loops. These movements correspond to the folding and unfolding of the loops that can influence the binding of co-regulators and co-chaperone complexes. Interestingly, AR bound to DHT fluctuates along the positive direction of PC2, which indicates the flexible H1–H3 and H8–H9 loops moves closer to H3 and H4, suggesting an increased stability of the AR. In PC3, the negative motion moves the H1–H3 and the H11–H12 loops outward, which characterizes the structural fluctuations observed from the WT AR bound to BCA. When combined, our PCA analysis showed significant differences in the conformational dynamics of WT AR bound to antagonists, agonist, and in *apo*.

**Figure 11:**
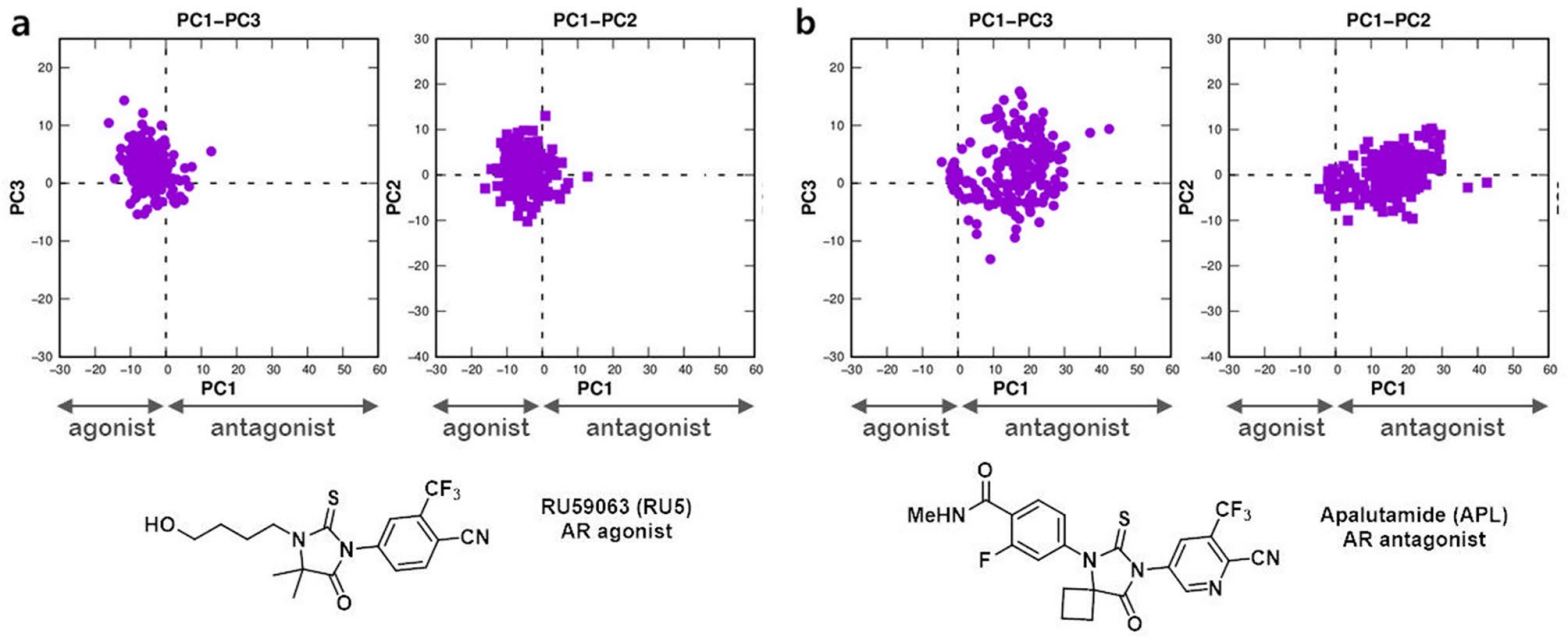
Projection of aMD trajectories of the wild type ARs bound to (**a**) RU5 and (**b**) APL in *conformation 2*. Images were created by using Gnuplot v5.2^57^ and Chemdraw.

We demonstrate the utility of PCA as a measure of the structure–activity relationship (SAR) of a ligand of AR. We plot the projections of aMD trajectories of AR bound to an agonist (RU5) and an antagonist (APL), as shown in Fig. 11. In a previous section, we showed the AR bound to RU5 exhibit reduced conformational fluctuations than the ARs bound to antagonist. The projection on the computed PCs confirmed the binding of RU5 minimal structural changes in AR (Fig. 11a). On the contrary, the projection of aMD trajectories of AR bound to APL showed that the ligand acts as an antagonist, that exhibited large fluctuations along the PC1 and PC2 (Fig. 11b), which resembles that of AR bound to ENZ.

## Conclusion

We have explored the conformational dynamics of the AR bound to the agonists (DHT and RU5), the antagonists (BCA, ENZ and APL), and in the absence of the ligand (*apo*). We carried out > 250 ns aMD simulations of the ligand bound AR LBD complexes and the AR LBD in *apo,* initiated from available agonistic conformations of AR LBD. With the aMD simulations, we were able to expedite the conformational changes in the AR LBDs and to observe the influence of ligands on AR LBD that provides insights into how antagonists destabilize the AR LBD. A large and differential repositioning of H12 manifests the influence of ENZ and APL to the WT AR. The N-methylbenzamide tail of ENZ and APL is a salient structural element that induces repulsive interactions with the bulky and hydrophobic side chains of H12, leading to the destabilization of H12. The drug resistant mutations (F876L and W741L) suppress the influence of the antagonists by stabilizing the agonistic conformation of the AR. The simulations of BCA bound to AR show less pronounced repositioning of H12 but rather significant fluctuations in the H1–H3 loop. These distinct conformational changes caused by the first and the second generation AR antagonists were observed only from the aMD simulations, but not from conventional MD simulations. PCA results account for the conformational dynamics obtained from the aMD trajectories of the AR LBDs. These findings indicate that aMD simulations can guide future drug discovery of new-generation AR antagonists.

Among the intriguing questions to be addressed in the future, the detailed interactions between the selective ligands and the AR LBDs are paramount. The detailed orientation of the tail group of ENZ and APL in the binding pocket of AR LBD remains elusive. Our computational analyses suggested the N-methylbenzamide group of ENZ and APL oriented toward H12 causes displacement of the helix, but we were not able to exclude the other possible orientation to occur in *vivo*. Future computational analyses including the comparisons of the binding free energies of the two orientations will give decisive information about these effects. The applicability of the PCA that we have introduced as a measure for the structure–activity relationship (SAR) of the ligands of AR will need further validations, which are underway in our lab.

## Methods

### Preparation of the ligands and proteins for MD simulations

The x-ray crystal structures of the AR LBD (2AXA and 2PIP) were retrieved from Protein Data Bank for the simulation. 2AXA is the structure of synthetic agonist (S1) bound wild type AR and 2PIP is co-crystalized with DHT and BF3 inhibitor (K10) and both were used as a starting structure. For the mutant AR, the residue 876 was mutated from phenylalanine to leucine using mutagenesis wizard in PyMOL. The structure of bicalutamide (BCA) was built based on the co-crystalized S1 by replacing the ether linkage with the sulfonyl group and the nitro group with cyano group. The other ligand structures were virtually generated since no structures of the AR bound to antagonists have been determined. Each ligand was optimized with density functional theory (DFT) at the B3LYP/6-31G* using Gaussian 09 software^46^. The optimized ligands were docked to the each PDB structure using Autodock Vina that uses a scoring function combined knowledge-based potentials and empirical approach to rank ligands docked to protein structures. Protein receptors and ligands were prepared using default criteria from AutoDock Vina^47^. Receptors were treated as rigid, and the ligands were treated as a flexible molecule with active rotatable bonds. To perform molecular docking, all hydrogen atoms were added to each protein and ligand of which coordinate files were generated as PDBQT file by merging nonpolar hydrogen atoms of the AR and calculating Gasteiger charges. The search space for docking was determined based on the three dimensional dimension of the original ligand from the crystal structure. A grid box for binding site was set as 20, 24 and 20 in the three dimensions (x, y and z centered at 26.3, 2.4 and 6.5, respectively) using 1.0 Å grid spacing. The predicted binding affinity of an agonist (FHM) was − 10.6 kcal/mol when we used 2AXA as the agonistic conformation. In each docking experiment, the pose with best calculated binding affinity (i.e. − 0.4 kcal/mol for *conformation 1* or − 0.8 kcal/mol for *conformation 2* for ENZ) was selected for further MD simulations. The docking simulations of the other ligands were performed with same condition as described above.

### MD simulations

Molecular mechanics parameters were constructed using the Amber14 software package^48^. The Amber FF99SB force field was used for the proteins^49^. The generalized Amber Force Field (GAFF) was used to parameterize the ligands (DHT, RU59063, bicalutamide (BCA), enzalutamide (ENZ) and apalutamide (APL)). Partial atomic charges on each ligand were determined by the quantum mechanical (QM) calculations based on the Merz-Singh-Kollman algorithm using Gaussian 09. Initially, each ligand is docked in the ligand binding pocket manually. Each protein and ligand complex was immersed in a rectangular solvation box filled TIP3P water molecules, with a 15 Å margin from the solvation boundary^50^. Monovalent counter ions (Na^+^ or Cl^−^) were added to achieve electrostatic neutrality of the systems. Each solvated system consists of approximately 48,000 atoms. The second step of the preparatory steps was to equilibrate the solvated system. The equilibration steps were initiated by energy minimizations and then equilibrating each solvated system at 300 K using the NVT ensemble for 2 ns. Berendsen thermostat was used to control the temperature at the desired value, and then the isobaric ensemble (NPT) was used for next 4 ns. Extended system algorithm was used to control the system pressure at 1 atm. The coordinates of the carbon atoms were restrained during the first 6 ns equilibration period. A 250 ns equilibration period followed, without any restraint of the atomic coordinates. Langevin thermostat with a small friction coefficient (5 ps^−1^) and a Monte Carlo barostat were used to keep the temperature at 300 K, and the pressure at 1 atm throughout the equilibration period. For the entire equilibration steps, the time integration step was 2 fs. The SHAKE algorithm was employed to restrain all hydrogens to heavy atoms bond distances. The particle mesh EWALD method accelerated the computation of long-range electrostatic interactions.

### Accelerated MD simulations

Accelerated MD enhances the conformational sampling of the protein complex by adding a non-negative boost potential to the system. We initially carried out 250 ns MD simulations of the LBDs bound to the ligands. The backbone root-mean-square deviation (RMSD) from the initial conformation were < 2.0 Å (Figure S1). Moreover, we were not able to identify conformational changes that are specific to each ligand considered. The observations prompted us to utilize aMD algorithm that is known to facilitate conformational changes otherwise difficult to sample from MD simulations. The Hamiltonian of a simulated system ($H\left(\overrightarrow{r}\right)$) is modified in such a way to elevate the local minimum via the boost potential ($\Delta \mathrm{V}\left(\overrightarrow{r}\right)$),

$${H}^{*}\left(\overrightarrow{r}\right)=H\left(\overrightarrow{r}\right)+\Delta \mathrm{V}\left(\overrightarrow{r}\right),$$

where ${H}^{*}\left(\overrightarrow{r}\right)$ is the modified Hamiltonian. The boost potential is defined as following:

$$\Delta V\left(\overrightarrow{r}\right)=\Delta {V}_{P}\left(\overrightarrow{r}\right)+\Delta {V}_{D}\left(\overrightarrow{r}\right)$$

$$\Delta {V}_{P}\left(\overrightarrow{r}\right)=\frac{{\left({E}_{P}-V\left(\overrightarrow{r}\right)\right)}^{2}}{\left(\alpha P+{E}_{P}-V\left(\overrightarrow{r}\right)\right)} \quad \text{and} \quad\Delta {V}_{D}\left(\overrightarrow{r}\right)=\frac{{\left({E}_{D}-{V}_{D}\left(\overrightarrow{r}\right)\right)}^{2}}{\left(\alpha D+{E}_{D}-{V}_{D}\left(\overrightarrow{r}\right)\right)},$$

where $\Delta {V}_{P}\left(\overrightarrow{r}\right)$ and $\Delta {V}_{D}\left(\overrightarrow{r}\right)$ are the boosting potentials of the total energy and the dihedral energy, respectively, ${E}_{P}$ is the total potential energy, $\alpha P$ is the control parameter of the boosting potential of the total potential energy, ${E}_{D}$ is the dihedral energy, and $\alpha D$ is the control parameter of the boosting potential of the dihedral energy. Of note, either of the boosting potentials ($\Delta {V}_{P}\left(\overrightarrow{r}\right)$ or $\Delta {V}_{D}\left(\overrightarrow{r}\right)$) is applied, when the total energy (${E}_{P}$) or the dihedral energy (${E}_{D}$) is lower than the threshold values: ${E}_{threshP}$ and ${E}_{threshD}$. We chose $\alpha P$ as to ensure each atom of a solvated system experience 0.2 kcal/mol of boosting potential and $\alpha D$ as to elevate 0.7 kcal/mol of dihedral energy of per each amino acid^51^. The four control parameters of the aMDs were determined as following.

$$\alpha P=0.2\times {N}_{atom}$$

$${E}_{threshP}=\alpha P+\overline{{E }_{P}}$$

$$\alpha D=0.7\times {N}_{res}$$

$${E}_{threshD}=3.5\times {N}_{res}+\overline{{E }_{D}}$$

where ${N}_{atom}$ is the number of atoms in a solvated system, ${N}_{res}$ is the number amino acids, and $\overline{{E }_{P}}$ and $\overline{{E }_{D}}$ are the total and the dihedral energies averaged over the initial 250 ns MD simulations.

Once MD simulations were finished, trajectories were analyzed using the AmberTools14 software package. The backbone root-mean square deviations (RMSDs) between the simulated aMD trajectories and the initial PDB structures and the root-mean square fluctuations (RMSFs) per residue over the time were computed using VMD and plugin^52^. We note that we have not attempted to construct potential energy profile of the conformational ensemble, as the original implementation of aMD methodology suffers from difficulty in reconstruction of the unbiased energy surface. In order to obtain proper conformational free energy surface of AR-antagonists systems, it is desirable to perform the other potential approaches such as Gaussian accelerated molecular dynamics (GaMD)^53^ for the future investigation.

### Principal component analysis

To understand conformational dynamics of AR bound to agonists and antagonists, principal component analysis (PCA) was performed with trajectories extracted from aMD simulations. With PCA, one can identify essential coordinates that describe the global motion of proteins^54^. Specifically, the diagonalization of interatomic correlation matrix results in characteristic modes of conformation motions. Each mode represents an orthogonal direction of global conformational motion of a protein, with an eigenvalue that quantifies the relative significance of the motion. A few large eigenvalue modes are of specific interest, as they represent significant conformational changes during the simulations. To process the aMD trajectories for PCA, the 250 ns MD trajectories of the ARs bound to the agonists and antagonists and *apo* AR were reoriented and aligned to the initial x-ray structure of the AR. PCA were performed for Cα atoms on snapshots at 250 ps intervals. MDAnalysis software^55^ was used to compute the first three principal components.

## Supplementary Information

Supplementary Information.
