# Supplementary material for: Conformational dynamics of androgen receptors bound to agonists and antagonists
Source: Sci Rep. 2021 Aug 5;11:15887. doi: 10.1038/s41598-021-94707-2 (PMC8342701; doi:10.1038/s41598-021-94707-2)
Supplement: Supplementary file 1 — Supplementary Information. [file 41598_2021_94707_MOESM1_ESM.pdf]

Supplementary Information

Conformational Dynamics of Androgen Receptors Bound to Agonists and Antagonists

Hyo Jin Gim[†], Jiyong Park[†], Michael E. Jung[*] and K. N. Houk[*]

Department of Chemistry and Biochemistry, University of California, Los Angeles, Los Angeles, California 90095-1569

jung@chem.ucla.edu Tel 310-825-7954

houk@chem.ucla.edu Tel 310-206-0515

[†] These authors contributed equally to this work

Table of Contents

Supplementary Figures 3
Supplementary Tables 15
Optimized Coordinates, RESP Charges, and Energies of the Ligands of AR 15
Bicalutamide 15
Enzalutamide 16
Apalutamide 17
RU59063 18
Dihydrotestosterone 19
DFT Computed Energies of the Ligands 21

Supplementary Figures

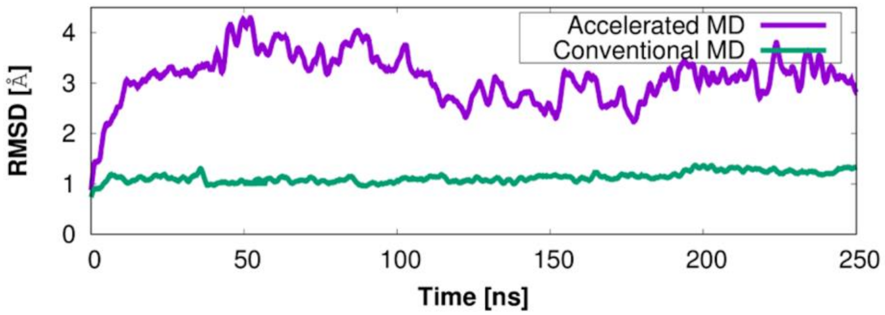

Figure S1. Comparison of the backbone RMSDs of the wild type AR bound to ENZ in *conformation 2*: conventional MD and accelerated MD simulations.

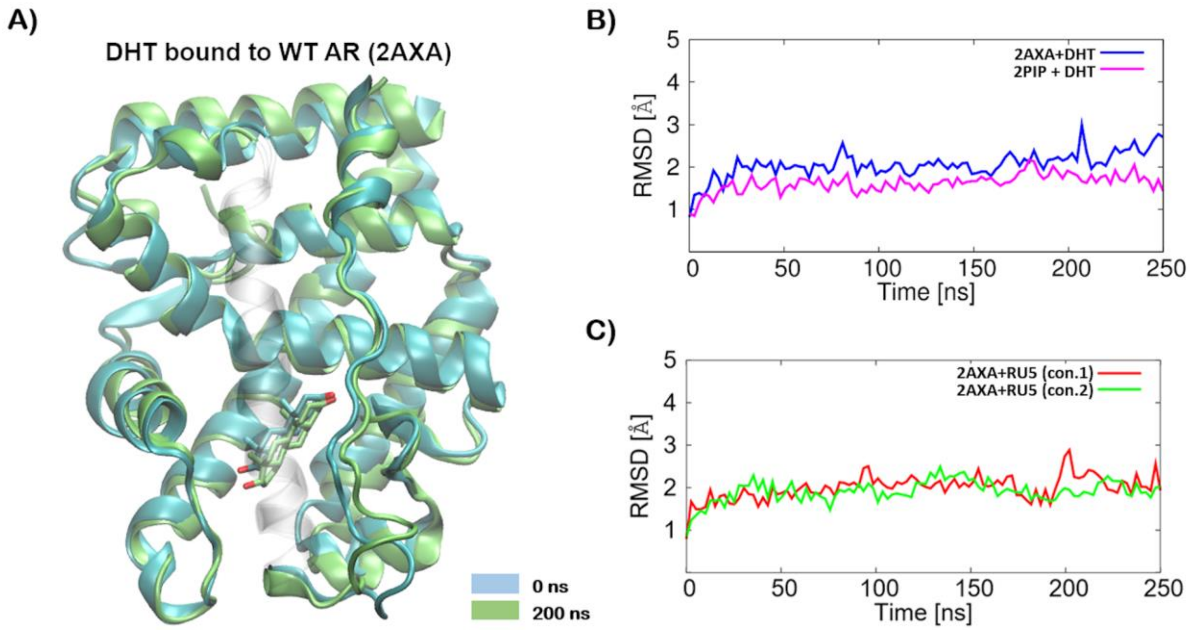

Figure S2. (A) The snapshots of DHT bound to the wild type AR of the initial structure (PDB ID: 2AXA) and aMD trajectories after 200 ns simulation. (B) The backbone RMSDs of the wild type ARs bound to DHT and (C) RU5 during 250 simulations.

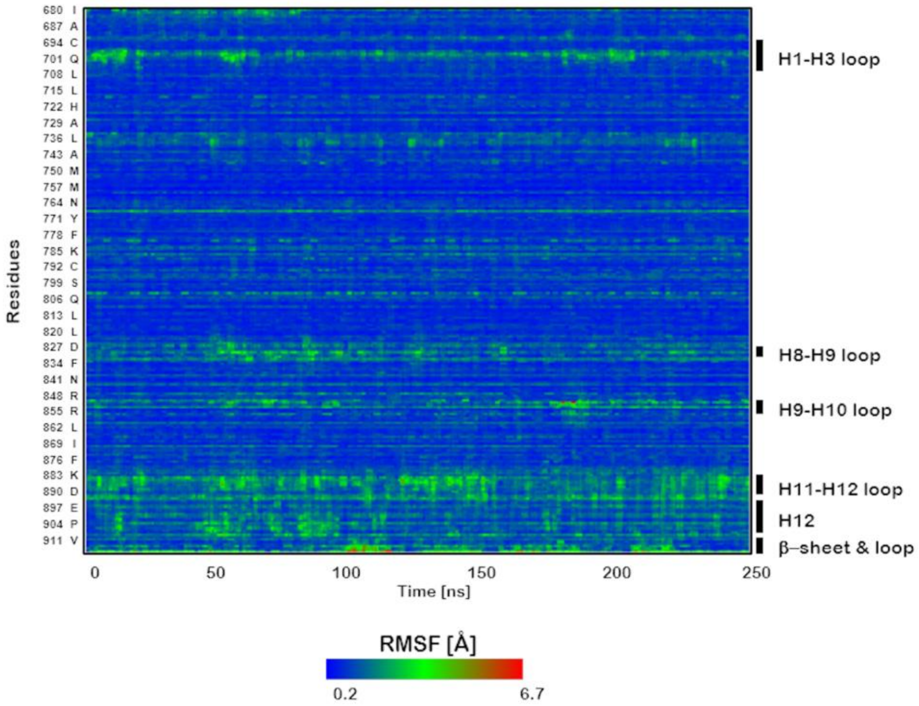

Figure S3. Cα RMSF over the time of the wild type AR bound to ENZ in *conformation 2*.

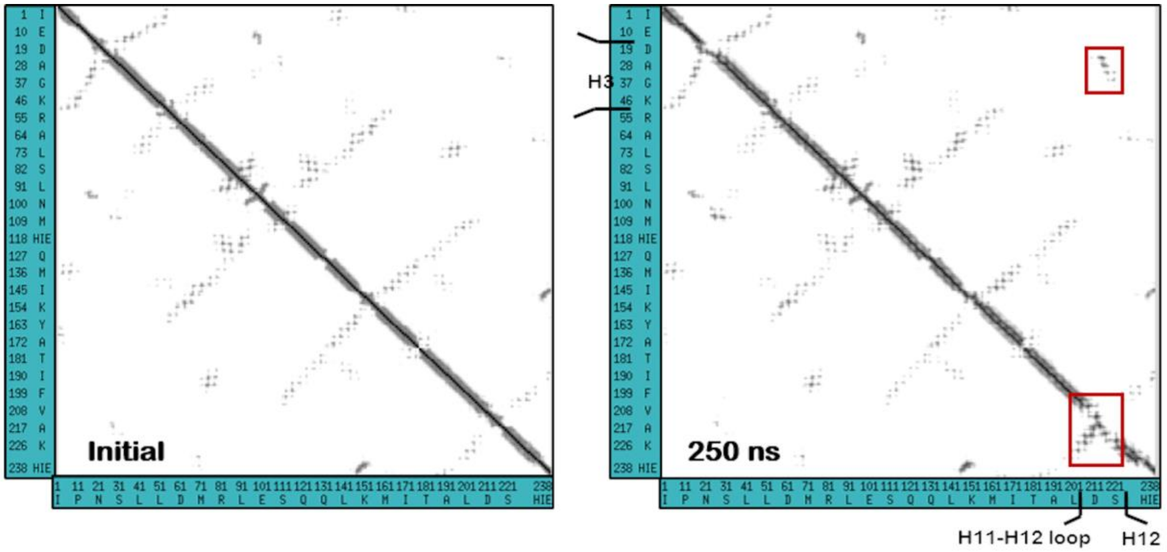

Figure S4. Cα to Cα distance of each residue pair in the initial (PDB ID: 2XAX) and simulated structures of the wild type AR bound to ENZ in *conformation 2*. A graph square is colored black at 0 Å, to a linear gray scale between 0 and 10 Å, and white when equal to or greater than 10 Å.

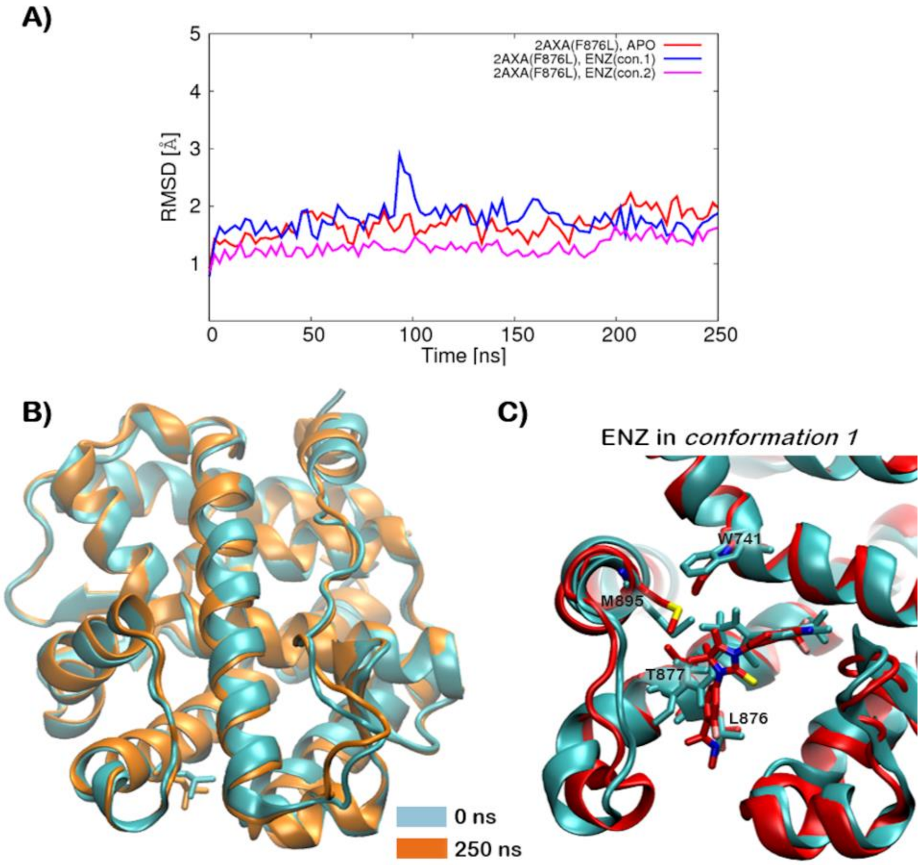

Figure S5. (A) The backbone RMSDs of the F876L mutant ARs bound to ENZ in *conformation 1, 2* and in the absence of the ligand. (B) A superimposed snapshot of the initial and 250 ns simulated structures of *apo* F876L mutant ARs. (C) The snapshot of F876L mutant AR bound to ENZ in *conformation 1.* The initial structures of F876L mutant ARs are colored in cyan and the simulated structures are colored in orange (*apo*) and red (ENZ in *conformation 1*), respectively.

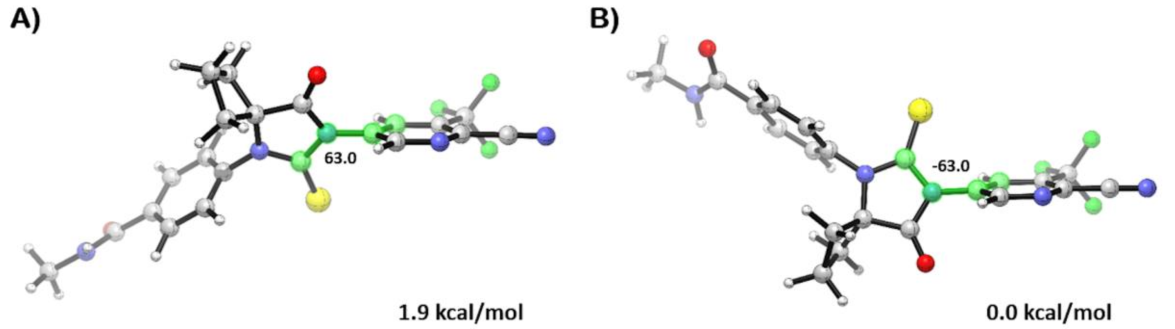

Figure S6. Two conformers of APL with the lowest energy. (A) Conformer with the second lowest energy (*conformation 1*) and (B) the other conformer with the lowest in energy conformation (*conformation 2*). The dihedral angles of the four highlighted atoms (C-C-N-C) are shown..

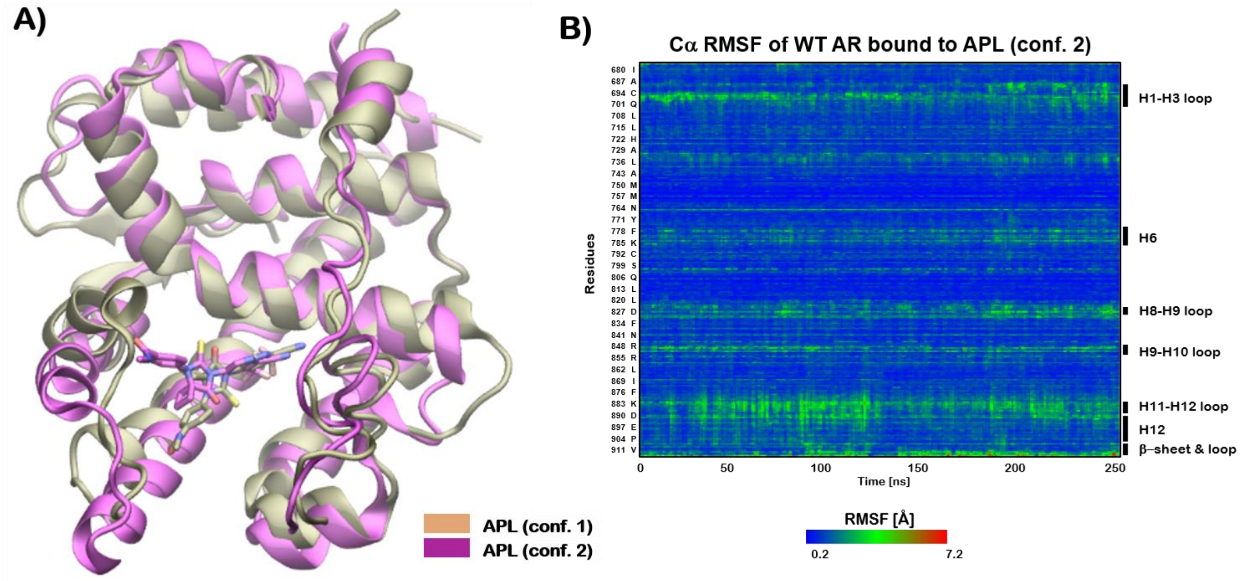

Figure S7. (A) The comparison of the wild type ARs bound to APL in *conformation1* and *conformations 2* after 250 ns aMD simulations. (B) Cα RMSF over the time during 250 ns aMD simulations (PDB ID: 2AXA).

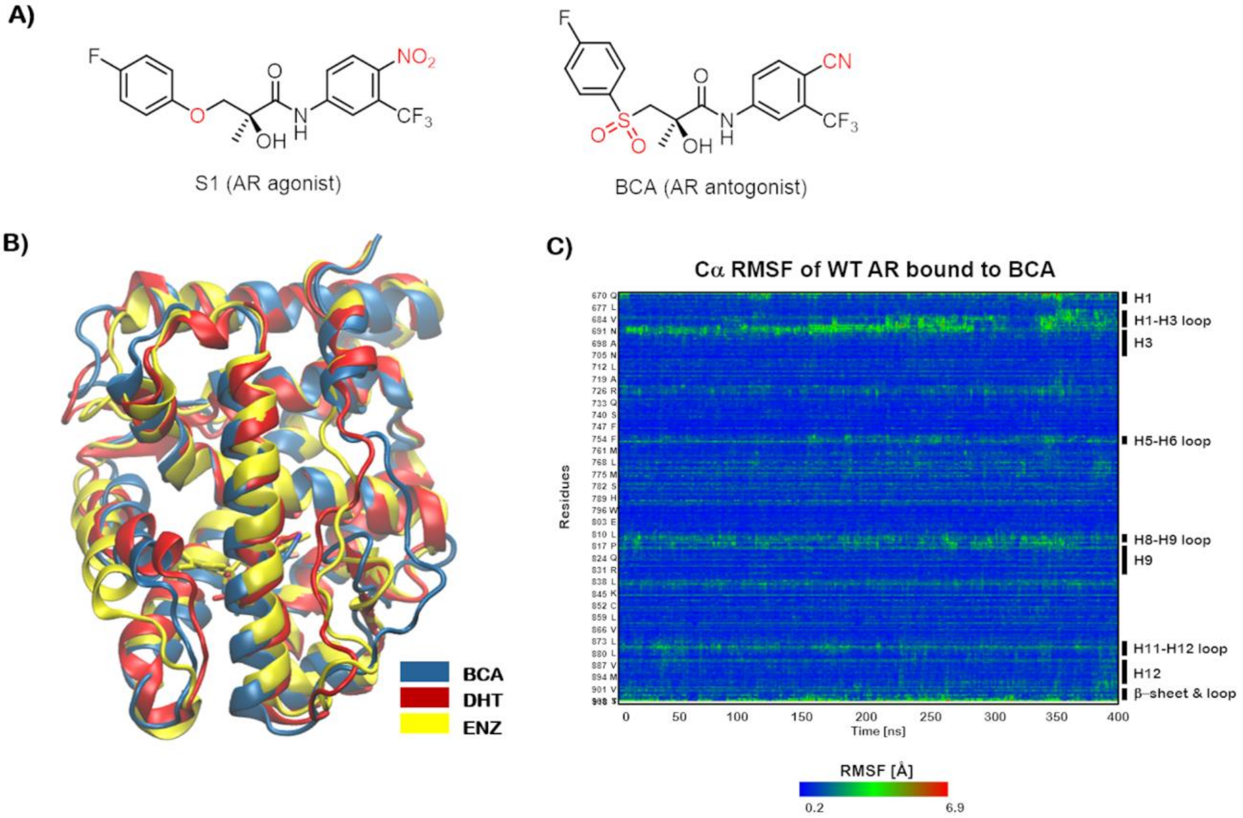

Figure S8. (A) Chemical structures of originally crystalized ligand S1 and BCA. (B) Comparison of the wild type ARs bound to BCA, DHT and ENZ after 250 ns aMD simulations. (C) Cα RMSF over the time during 400 ns aMD simulations (PDB ID: 2PIP).

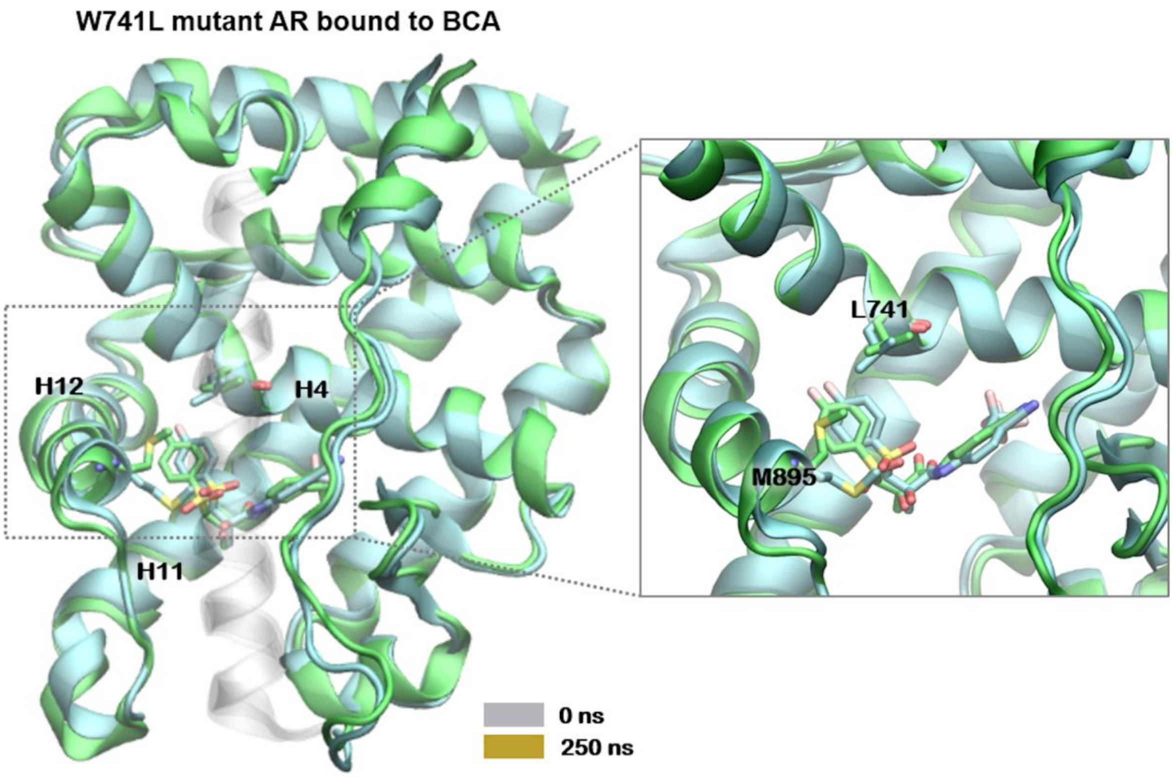

Figure S9. Comparison of W741L mutant ARs bound to BCA in *conformation 2* at 0 ns and 250 ns.

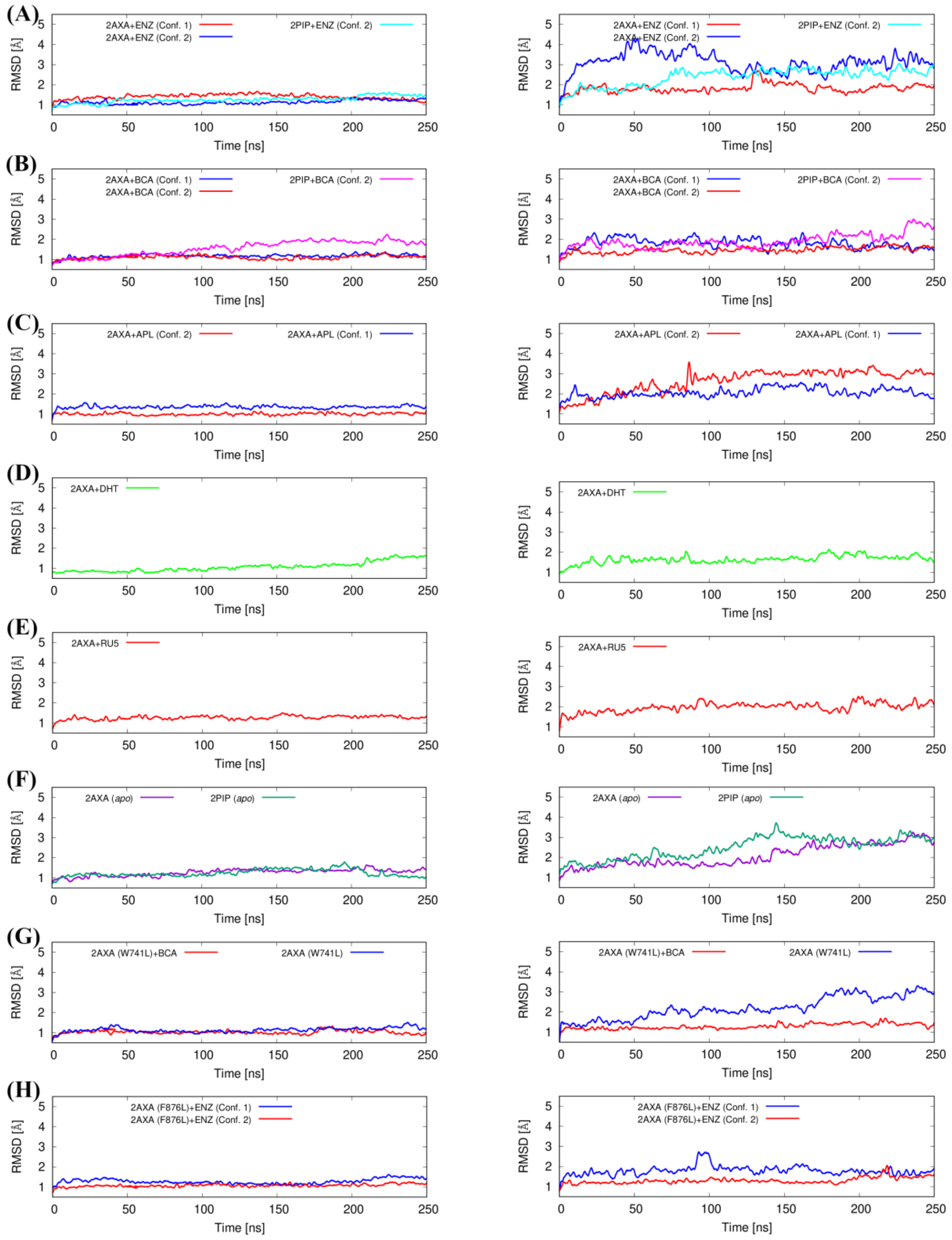

Figure S10. The backbone RMSDs of conventional MD simulations (left column) and aMD simulations (right column). (A) wild type ARs bound to ENZ (B) wild type ARs bound to BCA (C) wild type ARs bound to APL (D) wild type AR bound to DHT (E) wild type AR bound to RU5 (F) wild type ARs in apo (G) W741 mutant ARs bound to BCA (H) F876L mutant ARs bound to ENZ.

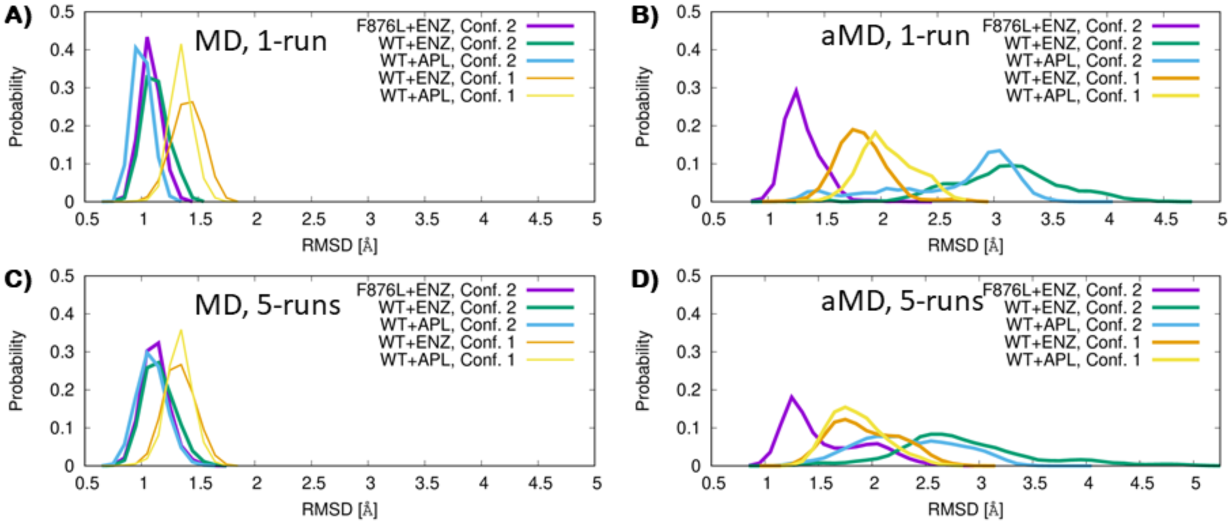

Figure S11. Comparison of the backbone RMSD sampled from single and multiple copies of MD and aMD simulations: probability density of RMSDs sampled from (A) single and (C) 5-copies of conventional MD; probability density of RMSDs sampled from (B) single and (D) 5-copies of accelerated MD simulations.

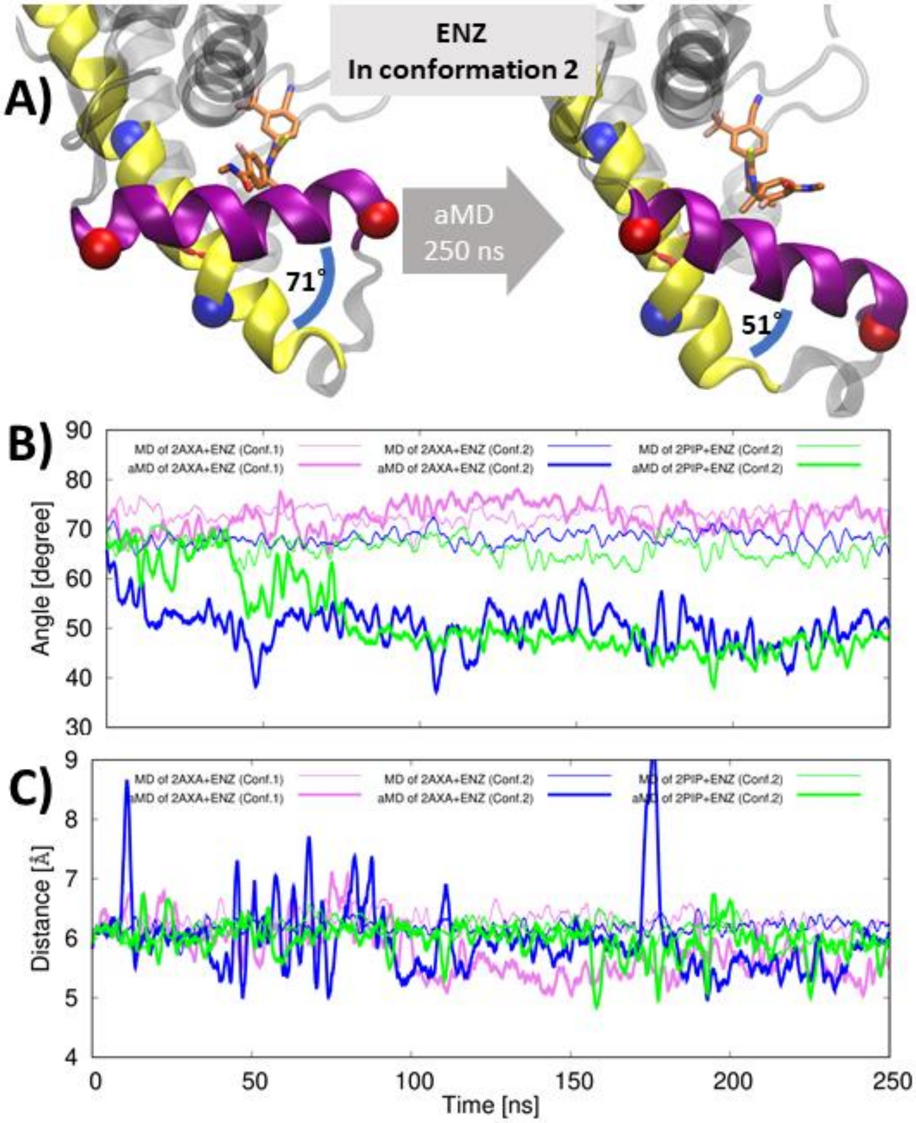

Figure S12. Conformational changes of the helix 11 (H11) in AR bound to enzalutamide, observed from the MD and the aMD simulations: (A) comparison of the H11 in the initial and the last (250 ns) conformation sampled from aMD simulation; (B) relative angles and (C) minimal distance between H11 and H12 observed from the MD and the aMD simulations.

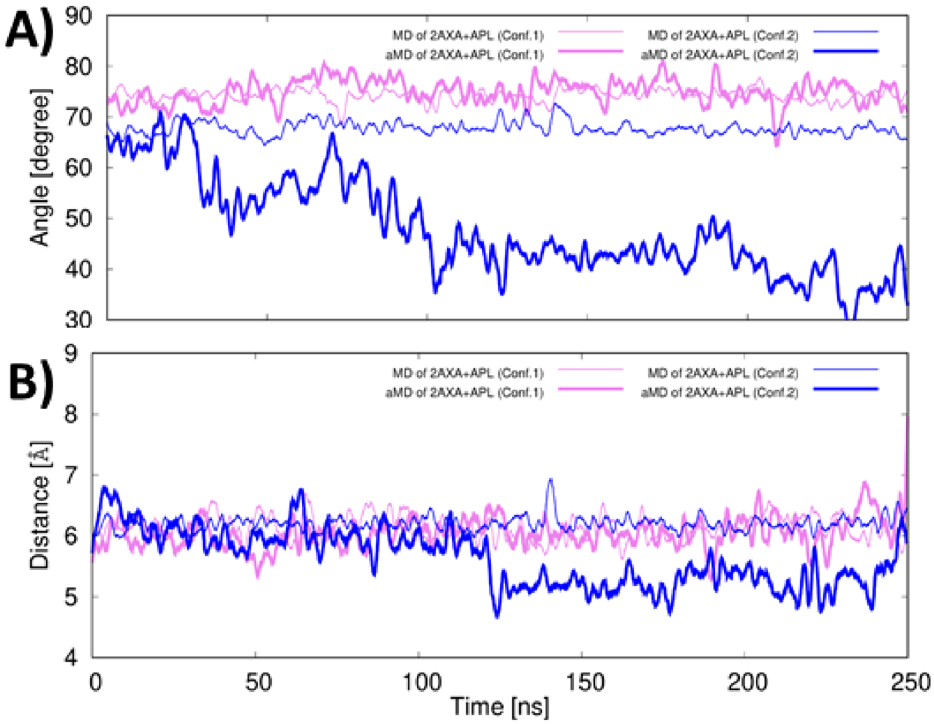

Figure S13. Conformational changes of the helix 11 (H11) in AR bound to apalutamide, observed from the MD and the aMD simulations: (A) relative angles and (B) minimal distance between H11 and H12 observed from the MD and the aMD simulations.

2AXA+ENZ (Conf. 1)		2AXA+ENZ (Conf. 2)	
**MD**	**aMD**	**MD**	**aMD**
Cluster 1 (15 %)	Cluster 1 (13 %)	Cluster 1 (25 %)	Cluster 1 (17 %)
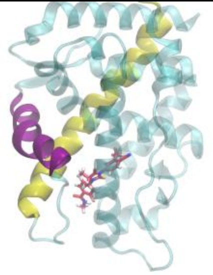	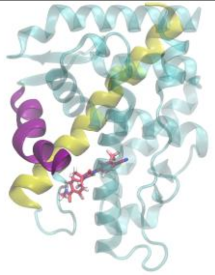	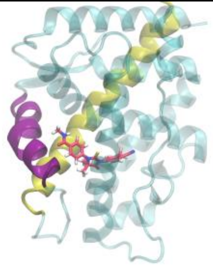	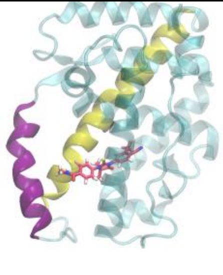
Cluster 2 (15 %)	Cluster 2 (13 %)	Cluster 2 (13 %)	Cluster 2 (14 %)
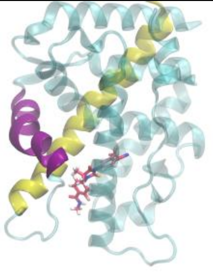	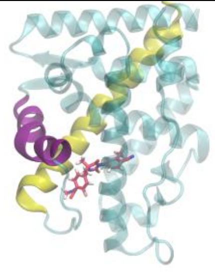	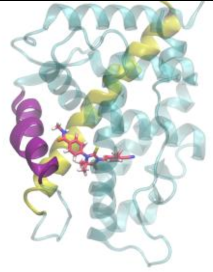	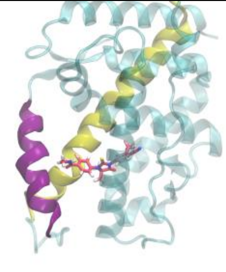

**2PIP+ENZ (Conf. 2)**		**2AXA in *apo***	
**MD**	**aMD**	**MD**	**aMD**
Cluster 1 (15 %)	Cluster 1 (14 %)	Cluster 1 (18 %)	Cluster 1 (19 %)
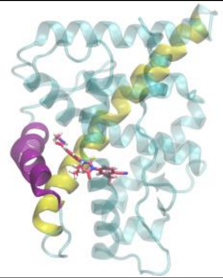	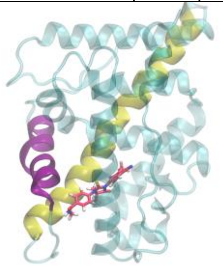	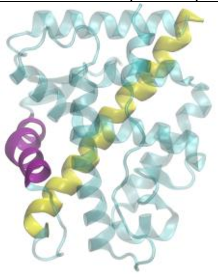	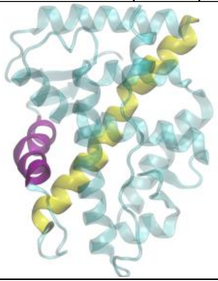
Cluster 2 (13 %)	Cluster 2 (13 %)	Cluster 2 (16 %)	Cluster 2 (13 %)
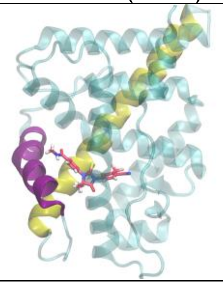	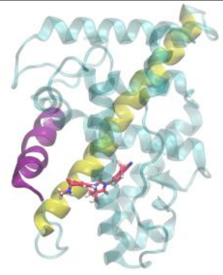	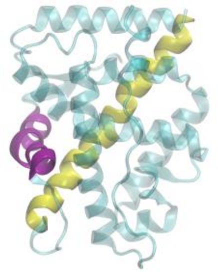	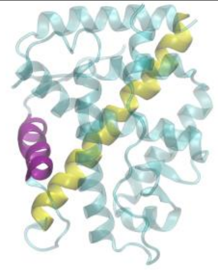
**2AXA+DHT**		**2PIP+DHT**	
**MD**	**aMD**	**MD**	**aMD**
Cluster 1 (15 %)	Cluster 1 (18 %)	Cluster 1 (15 %)	Cluster 1 (18 %)
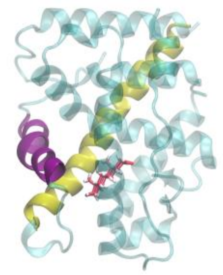	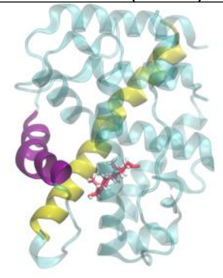	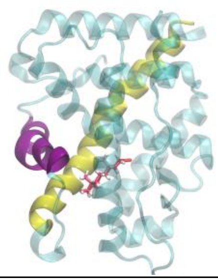	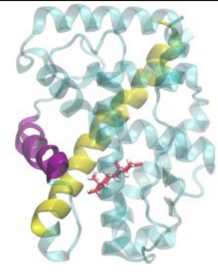
Cluster 2 (13 %)	Cluster 2 (14 %)	Cluster 2 (13 %)	Cluster 2 (14 %)
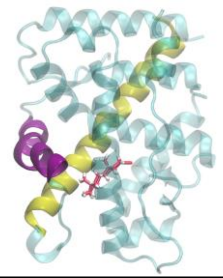	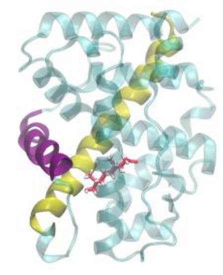	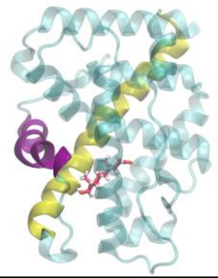	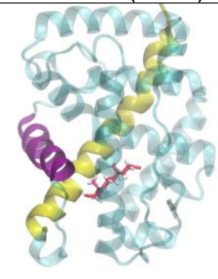
**2AXA+RU5 (Conf. 1)**		**2AXA+RU5 (Conf. 2)**	
**MD**	**aMD**	**MD**	**aMD**
Cluster 1 (15 %)	Cluster 1 (15 %)	Cluster 1 (17 %)	Cluster 1 (14 %)

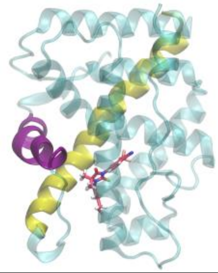	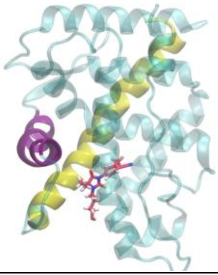	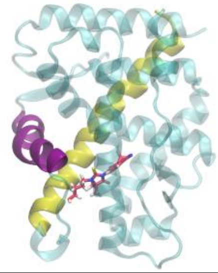	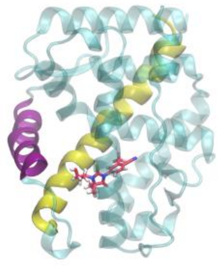
Cluster 2 (12 %)	Cluster 2 (13 %)	Cluster 2 (14 %)	Cluster 2 (14 %)
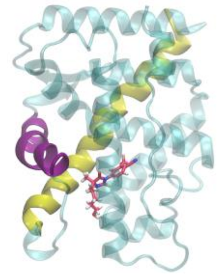	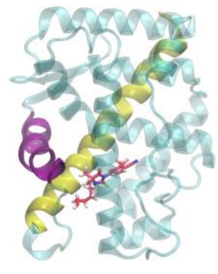	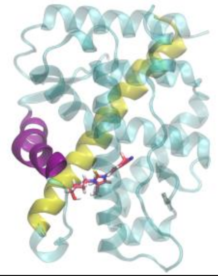	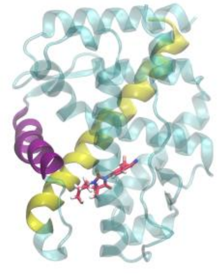
**2AXA(F876L)+ENZ**		**2AXA+APL (Conf. 1)**	
**MD**	**aMD**	**MD**	**aMD**
Cluster 1 (18 %)	Cluster 1 (14 %)	Cluster 1 (16 %)	Cluster 1 (18 %)
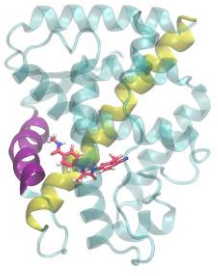	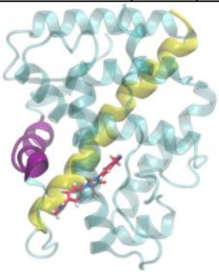	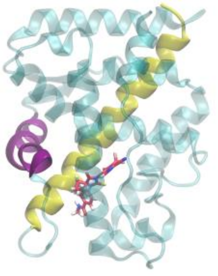	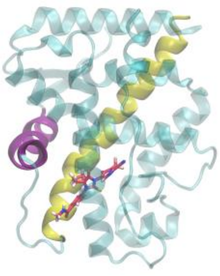
Cluster 2 (16 %)	Cluster 2 (13 %)	Cluster 2 (12 %)	Cluster 2 (13 %)
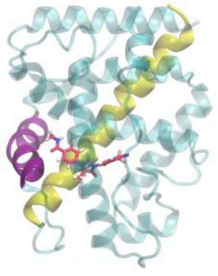	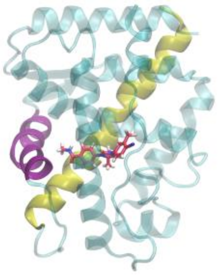	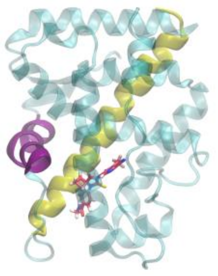	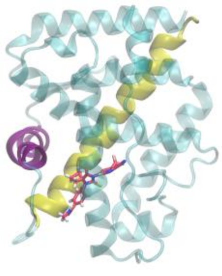
**2AXA+APL (Conf. 2)**		**2AXA+BCA**	
**MD**	**aMD**	**MD**	**aMD**
Cluster 1 (16 %)	Cluster 1 (15 %)	Cluster 1 (18 %)	Cluster 1 (15 %)
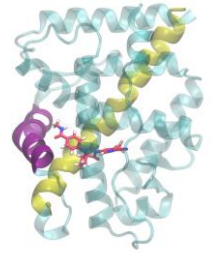	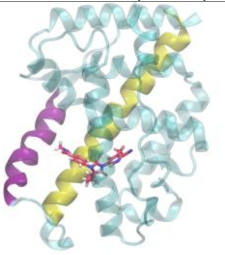	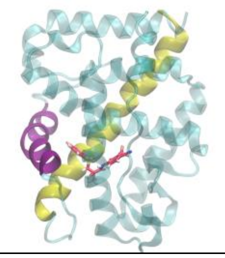	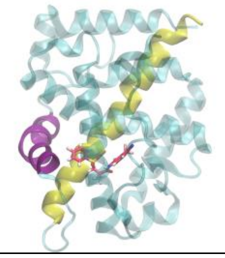
Cluster 2 (12 %)	Cluster 2 (13 %)	Cluster 2 (12 %)	Cluster 2 (13 %)

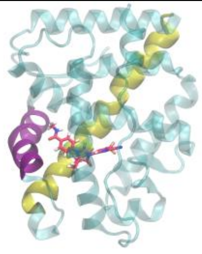	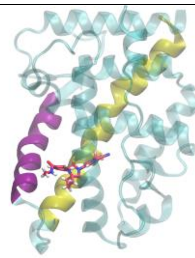	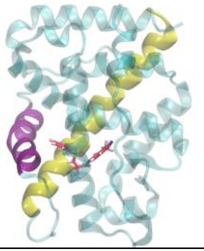	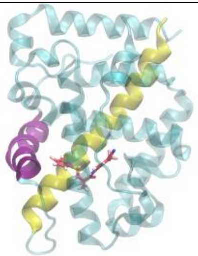
**2PIP+BCA**			
**MD**	**aMD**		
Cluster 1 (17 %)	Cluster 1 (15 %)		
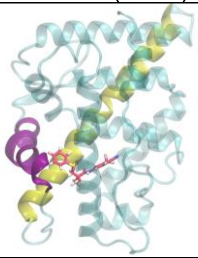	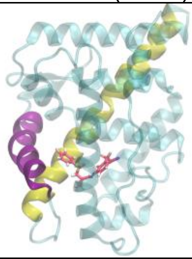		
Cluster 2 (12 %)	Cluster 2 (12 %)		
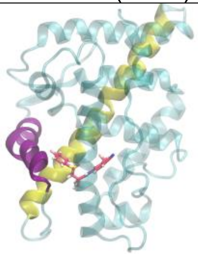	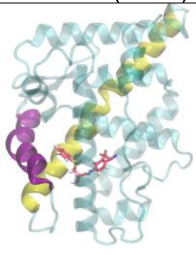		

Figure S14. Summary of structural clustering analysis of the MD and aMD trajectories. For each 250 ns MD and aMD simulation trajectories, we applied K-means clustering algorithm implemented in MDAnalysis that classified the conformational ensemble into 10 structural clusters. Backbone RMSD was used as the measure of structural similarities. Helix 11 and 12 are highlighted in purple and yellow, respectively. Ligands are color coded in red.

Supplementary Tables

Optimized Coordinates, RESP Charges, and Energies of the Ligands of AR

Bicalutamide

	Configuration 1				Configuration 2			Partial
**Atom**	X	Y	Z	**Atom**	X	Y	Z	**Charge**
**C**	52.703	48.479	42.262	**C**	8.791	1.658	7.152	-0.087060
**C**	53.571	49.427	41.735	**C**	9.361	0.444	7.511	-0.316968
**C**	53.027	50.574	41.171	**C**	8.966	-0.697	6.826	0.442890
**F**	53.865	51.495	40.660	**F**	9.517	-1.874	7.172	-0.220835
**C**	51.662	50.815	41.106	**C**	8.027	-0.683	5.803	-0.316968

C	50.799	49.862	41.636	C	7.460	0.534	5.446	-0.087060
C	51.329	48.707	42.208	C	7.848	1.688	6.124	-0.026580
C	50.141	47.997	44.651	C	8.337	3.914	4.452	-0.489743
C	49.484	46.956	45.595	C	7.989	5.341	3.989	0.477428
O	50.327	45.850	45.760	O	7.840	6.242	5.071	-0.657679
C	49.317	47.586	46.980	C	9.127	5.866	3.110	-0.481787
C	48.136	46.452	45.013	C	6.711	5.288	3.112	0.707155
O	48.056	45.315	44.579	O	6.657	4.545	2.143	-0.595838
N	47.111	47.354	45.010	N	5.739	6.154	3.506	-0.620068
C	45.850	47.227	44.414	C	4.468	6.341	2.961	0.386999
C	45.482	46.114	43.648	C	4.014	5.631	1.841	-0.213291
C	44.943	48.276	44.592	C	3.627	7.275	3.575	-0.323835
C	43.687	48.220	44.013	C	2.355	7.503	3.080	-0.092118
C	43.309	47.114	43.246	C	1.894	6.801	1.961	-0.044519
C	44.223	46.062	43.071	C	2.739	5.862	1.349	-0.060682
C	43.841	44.855	42.245	C	2.269	5.090	0.138	0.613358
F	42.746	44.245	42.741	F	1.158	4.375	0.407	-0.200591
F	43.558	45.197	40.973	F	1.966	5.915	-0.885	-0.200591
F	44.818	43.938	42.200	F	3.193	4.228	-0.311	-0.200591
H	53.091	47.564	42.698	H	9.066	2.569	7.672	0.185333
H	54.644	49.284	41.751	H	10.094	0.374	8.306	0.203999
H	51.291	51.722	40.645	H	7.749	-1.605	5.306	0.203999
H	49.726	50.008	41.590	H	6.715	0.584	4.660	0.185333
H	49.617	48.956	44.651	H	8.359	3.233	3.598	0.181527
H	51.170	48.149	44.985	H	9.304	3.907	4.960	0.181527
H	50.165	45.235	45.030	H	7.582	5.762	5.877	0.399013
H	50.309	47.779	47.396	H	10.057	5.874	3.682	0.143094
H	48.774	48.534	46.958	H	9.247	5.247	2.219	0.143094
H	48.796	46.890	47.641	H	8.900	6.890	2.801	0.143094
H	46.181	45.304	43.508	H	4.662	4.907	1.370	0.213813
H	45.218	49.142	45.185	H	3.969	7.824	4.447	0.198380
H	42.988	49.036	44.155	H	1.708	8.227	3.560	0.176029
S	50.224	47.501	42.912	S	7.148	3.243	5.636	0.878336
O	50.864	46.186	42.832	O	7.180	4.154	6.797	-0.534570
O	48.894	47.697	42.329	O	5.873	3.002	4.963	-0.534570
C	41.996	47.102	42.666	C	0.568	7.073	1.482	0.351347
N	40.929	47.129	42.213	N	-0.507	7.320	1.124	-0.449811
H	47.289	48.250	45.440	H	5.988	6.704	4.320	0.340009

Enzalutamide

	Conformation 1				Conformation 2			Partial Charge
**Atom**	X	Y	Z	**Atom**	X	Y	Z	
C	4.205	5.459	3.414	C	4.236	5.409	3.423	0.051961
C	3.732	4.887	2.235	C	3.614	5.258	2.188	-0.179428
C	3.496	6.460	4.062	C	3.716	6.256	4.392	-0.174677
C	2.295	6.904	3.523	C	2.544	6.953	4.128	-0.109482
C	1.814	6.350	2.336	C	1.906	6.810	2.893	0.008209
C	2.540	5.334	1.689	C	2.449	5.959	1.916	-0.032321
C	2.027	4.723	0.405	C	1.772	5.793	0.575	0.607718
F	0.817	4.156	0.576	F	0.527	5.299	0.708	-0.196866
F	1.897	5.647	-0.565	F	1.663	6.968	-0.073	-0.196866
F	2.849	3.770	-0.061	F	2.447	4.957	-0.229	-0.196866
H	4.301	4.103	1.751	H	4.041	4.593	1.448	0.181595

**H**	3.879	6.893	4.978	**H**	4.216	6.363	5.347	0.178307
**H**	1.730	7.686	4.016	**H**	2.121	7.614	4.875	0.175781
**C**	0.571	6.848	1.810	**C**	0.696	7.553	2.665	0.324590
**N**	-0.432	7.273	1.415	**N**	-0.277	8.165	2.516	-0.422226
**C**	6.951	4.004	5.455	**C**	7.666	3.977	3.528	0.317912
**N**	5.423	4.992	3.982	**N**	5.443	4.695	3.669	0.183156
**C**	5.481	4.287	5.175	**C**	6.636	4.980	3.023	0.380350
**O**	4.533	3.965	5.855	**O**	6.810	5.861	2.213	-0.501238
**C**	6.696	5.217	3.443	**C**	5.562	3.602	4.537	-0.288105
**S**	7.010	6.053	2.048	**S**	4.334	2.963	5.448	-0.260448
**N**	7.579	4.636	4.280	**N**	6.844	3.191	4.467	0.300095
**C**	7.375	4.687	6.758	**C**	8.170	3.120	2.364	-0.463683
**C**	7.199	2.494	5.496	**C**	8.808	4.705	4.239	-0.463683
**H**	8.438	4.513	6.940	**H**	8.885	2.378	2.729	0.136468
**H**	7.195	5.765	6.715	**H**	7.345	2.603	1.868	0.136468
**H**	6.804	4.266	7.588	**H**	8.673	3.761	1.638	0.136468
**H**	8.258	2.296	5.682	**H**	9.540	3.982	4.609	0.136468
**H**	6.614	2.057	6.308	**H**	9.305	5.372	3.530	0.136468
**H**	6.908	2.021	4.555	**H**	8.436	5.297	5.079	0.136468
**C**	8.992	4.694	4.103	**C**	7.357	2.073	5.188	-0.007598
**C**	9.690	5.803	4.568	**C**	7.259	0.803	4.632	-0.287575
**C**	9.664	3.637	3.496	**C**	7.974	2.262	6.422	-0.254297
**C**	11.064	5.829	4.412	**C**	7.788	-0.265	5.335	0.361535
**H**	9.182	6.640	5.033	**H**	6.773	0.638	3.678	0.190575
**C**	11.045	3.697	3.363	**C**	8.498	1.167	7.094	0.033293
**H**	9.110	2.784	3.124	**H**	8.035	3.255	6.852	0.166275
**C**	11.785	4.793	3.817	**C**	8.420	-0.128	6.571	-0.298023
**H**	11.591	2.886	2.896	**H**	8.985	1.290	8.054	0.143412
**F**	11.704	6.930	4.879	**F**	7.669	-1.486	4.758	-0.222517
**C**	13.282	4.730	3.612	**C**	9.035	-1.232	7.403	0.728769
**O**	13.791	3.714	3.138	**O**	9.655	-0.950	8.428	-0.586797
**N**	14.020	5.802	3.955	**N**	8.876	-2.504	6.993	-0.488388
**H**	13.571	6.621	4.332	**H**	8.356	-2.700	6.153	0.351799
**C**	15.459	5.807	3.764	**C**	9.432	-3.606	7.757	-0.285454
**H**	15.921	4.973	4.298	**H**	10.509	-3.478	7.886	0.137466
**H**	15.710	5.717	2.704	**H**	8.971	-3.667	8.747	0.137466
**H**	15.856	6.746	4.150	**H**	9.242	-4.533	7.215	0.137466

Apalutamide

**Conformer 1**				**Conformer 2**				**Partial Charge**
**Atom**	X	Y	Z	**Atom**	X	Y	Z	
**C**	-2.390	0.560	-0.385	**C**	3.877	5.702	2.718	-0.256829
**C**	-3.058	-0.376	0.392	**C**	2.631	5.099	2.631	0.099050
**C**	-3.069	1.218	-1.407	**C**	3.987	7.079	2.541	0.358040
**N**	-4.354	0.986	-1.676	**N**	2.936	7.855	2.290	-0.585608
**C**	-4.994	0.078	-0.938	**C**	1.735	7.277	2.215	0.569683
**C**	-4.392	-0.628	0.111	**C**	1.527	5.902	2.380	-0.362097
**C**	-5.170	-1.638	0.926	**C**	0.147	5.294	2.273	0.672007
**F**	-6.235	-1.072	1.524	**F**	-0.686	5.795	3.201	-0.204484
**F**	-5.629	-2.642	0.158	**F**	-0.404	5.534	1.070	-0.204484
**F**	-4.414	-2.179	1.892	**F**	0.181	3.963	2.440	-0.204484
**H**	-2.550	-0.888	1.199	**H**	2.524	4.029	2.758	0.159698

**H**	-2.562	1.952	-2.024	**H**	4.954	7.567	2.603	0.108896
**C**	-6.380	-0.130	-1.297	**C**	0.638	8.183	1.946	0.208906
**N**	-7.491	-0.285	-1.591	**N**	-0.232	8.915	1.731	-0.376482
**C**	0.892	2.055	0.429	**C**	7.115	3.920	2.626	0.241445
**N**	-1.033	0.876	-0.140	**N**	5.044	4.932	2.944	0.066725
**C**	-0.610	2.134	0.280	**C**	6.082	4.842	2.021	0.480404
**O**	-1.335	3.083	0.485	**O**	6.118	5.402	0.949	-0.497923
**C**	0.046	-0.010	-0.294	**C**	5.299	4.169	4.093	-0.033217
**S**	-0.075	-1.582	-0.799	**S**	4.299	4.052	5.408	-0.288007
**N**	1.158	0.678	0.039	**N**	6.498	3.582	3.903	-0.059705
**C**	1.639	3.225	-0.269	**C**	7.580	2.797	1.662	-0.222753
**C**	1.405	2.593	1.795	**C**	8.555	4.498	2.584	-0.222753
**C**	2.472	0.123	0.014	**C**	7.114	2.707	4.848	0.120609
**C**	3.244	0.238	-1.140	**C**	6.866	1.340	4.775	-0.187461
**C**	2.969	-0.513	1.149	**C**	7.970	3.227	5.816	-0.187461
**C**	4.538	-0.265	-1.147	**C**	7.494	0.481	5.671	-0.057629
**H**	2.823	0.701	-2.027	**H**	6.173	0.958	4.033	0.151698
**C**	4.260	-1.021	1.130	**C**	8.589	2.365	6.712	-0.057629
**H**	2.345	-0.609	2.029	**H**	8.135	4.298	5.871	0.151698
**C**	5.059	-0.890	-0.009	**C**	8.367	0.988	6.637	-0.210990
**H**	4.663	-1.531	2.003	**H**	9.249	2.750	7.481	0.135122
**H**	5.128	-0.196	-2.058	**H**	7.268	-0.580	5.625	0.135122
**C**	6.448	-1.462	0.034	**C**	9.068	0.114	7.642	0.769186
**O**	6.714	-2.450	0.719	**O**	9.396	0.551	8.745	-0.572962
**N**	7.385	-0.826	-0.718	**N**	9.314	-1.162	7.271	-0.568693
**H**	7.161	0.062	-1.140	**H**	9.170	-1.434	6.311	0.322294
**C**	8.762	-1.282	-0.746	**C**	9.991	-2.089	8.158	-0.137092
**H**	9.227	-1.205	0.247	**H**	11.031	-1.792	8.328	0.102635
**H**	8.808	-2.328	-1.073	**H**	9.481	-2.125	9.122	0.102635
**H**	9.318	-0.660	-1.458	**H**	9.971	-3.081	7.705	0.102635
**C**	1.725	3.938	1.100	**C**	8.812	3.662	1.309	0.011207
**H**	2.311	2.058	2.088	**H**	9.132	4.130	3.436	0.085995
**H**	0.691	2.586	2.616	**H**	8.642	5.584	2.522	0.085995
**H**	2.682	4.397	1.350	**H**	9.770	3.145	1.251	0.042535
**H**	0.924	4.664	1.231	**H**	8.654	4.244	0.400	0.042535
**H**	1.111	3.720	-1.084	**H**	6.886	2.526	0.863	0.085995
**H**	2.624	2.890	-0.612	**H**	7.856	1.905	2.228	0.085995

RU59063

Atom	X	Y	Z	Partial Charge
**C**	-2.188	1.875	-0.754	-0.159377
**N**	-6.629	0.050	-0.629	-0.423424
**O**	-0.085	3.084	1.211	-0.518951
**S**	0.844	-0.894	-1.681	-0.298643
**C**	-3.551	1.618	-0.836	-0.100753
**N**	0.048	1.184	-0.111	0.151689
**C**	-4.068	0.399	-0.393	-0.002819
**N**	2.241	0.976	-0.326	0.237912
**C**	-3.203	-0.567	0.150	-0.009725
**C**	-1.845	-0.303	0.240	-0.164194
**C**	-1.343	0.911	-0.221	-0.003820
**C**	-3.741	-1.889	0.646	0.589287

**C**	-5.484	0.181	-0.512	0.329816
**C**	2.074	2.180	0.492	0.199879
**C**	0.556	2.253	0.611	0.485847
**C**	1.074	0.418	-0.699	-0.188296
**C**	2.702	2.031	1.88	-0.437683
**C**	2.579	3.436	-0.228	-0.437683
**F**	-2.766	-2.669	1.144	-0.195690
**F**	-4.649	-1.715	1.624	-0.195690
**F**	-4.340	-2.578	-0.339	-0.195690
**H**	-1.786	2.820	-1.099	0.174794
**H**	-4.224	2.361	-1.248	0.173601
**H**	-1.173	-1.043	0.654	0.174336
**H**	2.444	2.901	2.489	0.136732
**H**	2.337	1.130	2.380	0.136732
**H**	3.791	1.972	1.797	0.136732
**H**	2.292	4.319	0.347	0.136732
**H**	3.669	3.420	-0.316	0.136732
**H**	2.147	3.511	-1.230	0.136732
**C**	3.539	0.446	-0.715	-0.394540
**H**	3.478	0.147	-1.766	0.132834
**H**	4.265	1.261	-0.642	0.132834
**C**	3.985	-0.742	0.139	0.029978
**H**	4.057	-0.431	1.189	0.004581
**H**	3.214	-1.518	0.080	0.004581
**C**	5.326	-1.296	-0.337	0.082329
**H**	6.104	-0.525	-0.275	0.035469
**H**	5.255	-1.596	-1.389	0.035469
**C**	5.771	-2.498	0.478	0.204708
**H**	5.849	-2.220	1.541	0.002094
**H**	5.021	-3.298	0.394	0.002094
**O**	7.026	-2.924	-0.022	-0.718761
**H**	7.305	-3.713	0.450	0.441214

Dihydrotestosterone

Atom	X	Y	Z	Partial Charge
**C**	2.522	-1.523	-0.436	0.052432
**C**	4.026	-1.486	-0.128	-0.249394
**C**	4.640	-0.121	-0.360	0.661308
**O**	5.706	0.023	-0.925	-0.571579
**C**	3.844	1.052	0.170	-0.448545
**C**	2.349	0.951	-0.170	0.301034
**C**	1.572	2.157	0.359	-0.210028
**C**	0.122	2.125	-0.118	-0.101511
**C**	-0.574	0.808	0.233	0.038721
**C**	0.243	-0.412	-0.266	-0.016783
**C**	1.714	-0.403	0.256	0.061847
**C**	-0.497	-1.741	-0.018	0.001121
**C**	-1.943	-1.742	-0.543	-0.372913
**C**	-2.734	-0.552	0.003	0.039673

C	-1.976	0.736	-0.364	0.074313
C	-2.983	1.860	-0.075	-0.317119
C	-4.360	1.219	-0.389	-0.043855
C	-4.075	-0.262	-0.706	0.488893
O	-5.178	-1.053	-0.303	-0.769669
C	-2.975	-0.709	1.512	-0.281744
C	1.759	-0.610	1.781	-0.441036
H	2.139	-2.506	-0.144	0.002633
H	2.378	-1.440	-1.522	0.002633
H	4.193	-1.735	0.929	0.075237
H	4.576	-2.218	-0.724	0.075237
H	3.981	1.086	1.260	0.114584
H	4.276	1.971	-0.236	0.114584
H	2.274	0.985	-1.269	-0.012647
H	1.609	2.171	1.457	0.061152
H	2.058	3.080	0.021	0.061152
H	-0.435	2.966	0.311	0.035775
H	0.100	2.258	-1.210	0.035775
H	-0.666	0.760	1.328	0.048034
H	0.316	-0.293	-1.361	0.004315
H	-0.505	-1.968	1.054	0.043298
H	0.044	-2.562	-0.500	0.043298
H	-2.424	-2.694	-0.280	0.098011
H	-1.925	-1.684	-1.641	0.098011
H	-1.840	0.709	-1.460	0.008858
H	-2.922	2.172	0.974	0.090598
H	-2.790	2.750	-0.681	0.090598
H	-5.038	1.264	0.468	0.033708
H	-4.869	1.708	-1.224	0.033708
H	-3.918	-0.385	-1.790	-0.035722
H	-5.055	-1.949	-0.630	0.431203
H	-3.582	-1.600	1.700	0.076758
H	-2.040	-0.814	2.067	0.076758
H	-3.516	0.141	1.935	0.076758
H	1.458	-1.629	2.040	0.106842
H	1.097	0.076	2.315	0.106842
H	2.764	-0.463	2.187	0.106842

DFT Computed Energies of the Ligands

LIGAND	CONF.	E(SCF)*	ZPE	-TS(GAS)	ΔG	ΔΔG
**BICALUTAMIDE**	1	-51752.502	192.12	-55.00	-1193286.39	5.61
	2	-51752.770	192.53	-54.54	-1193292.00	0.00
**ENZALUTAMIDE**	1	-53783.860	219.18	-58.78	-1240105.66	1.47
	2	-53783.860	218.97	-58.78	-1240107.13	0.00
**APALUTAMIDE**	1	-52555.982	221.11	-65.92	-1211788.24	1.86
	2	-52555.980	221.02	-60.19	-1211790.10	0.00
**RU59063**		-45463.81	213.22	-53.50	-1048243.63	
**DHT**		-24283.24	293.79	-41.12	-559719.37	

For bicalutamide, enzalutamide and apalutamide, geometries were optimized at the ωB97X-D/6-31+G(d,p)/IEFPCM(diethylether) level of the theory. For RU59063 and dihydrotestosterone (DHT), the geometries were obtained using the ωB97X-D/6-31+G(d,p) level of the theory. Vibrational frequencies of the optimized geometries were computed to obtain thermal corrections to the Gibbs free energies including zero-point energy (ZPE) and entropic contribution (-TS). The Gibbs free energy of molecule (ΔG) is the sum of E(SCF), ZPE, -TS, and the thermal corrections. Energy unit is kcal/mol, except for the SCF energy (E(SCF) that is in eV. Entropic contribution in gas (-TS(gas)) phase is measured at 298.15 K.
